# Laparoscopic vs Open Distal Gastrectomy for Locally Advanced Gastric Cancer: 5-Year Outcomes of the KLASS-02 Randomized Clinical Trial

**DOI:** 10.1001/jamasurg.2022.2749

**Published:** 2022-07-20

**Authors:** Sang-Yong Son, Hoon Hur, Woo Jin Hyung, Young-Kyu Park, Hyuk-Joon Lee, Ji Yeong An, Wook Kim, Hyoung-Il Kim, Hyung-Ho Kim, Seung Wan Ryu, Min-Chan Kim, Seong-Ho Kong, Gyu Seok Cho, Jin-Jo Kim, Do Joong Park, Keun Won Ryu, Young Woo Kim, Jong Won Kim, Joo-Ho Lee, Han-Kwang Yang, Sang-Uk Han

**Affiliations:** 1 Department of Surgery, Ajou University School of Medicine, Suwon, South Korea; 2 Department of Surgery, Yonsei University College of Medicine, Seoul, South Korea; 3 Department of Surgery, Chonnam National University Hwasun Hospital, Gwangju, South Korea; 4 Department of Surgery, Seoul National University Hospital, Seoul, South Korea; 5 Department of Surgery, Samsung Medical Center, Sungkyunkwan University School of Medicine, Seoul, South Korea; 6 Department of Surgery, Yeouido St Mary’s Hospital, The Catholic University of Korea, Seoul, South Korea; 7 Department of Surgery, Seoul National University Bundang Hospital, Seongnam, South Korea; 8 Keimyung University Dongsan Medical Center, Daegu, South Korea; 9 Department of Surgery, Dong-A University Hospital, Korea, Busan, South Korea; 10 Department of Surgery, Soonchunhyang University Bucheon Hospital, Bucheon, South Korea; 11 Department of Surgery, Incheon St Mary’s Hospital, The Catholic University of Korea, Incheon, South Korea; 12 Center for Gastric Cancer, National Cancer Center, Goyang, South Korea; 13 Department of Surgery, Chung-Ang University Hospital, Seoul, South Korea; 14 Department of Surgery, Nowon Eulji Medical Center, Eulji University, Seoul, South Korea

## Abstract

**Importance:**

The long-term safety of laparoscopic distal gastrectomy for locally advanced gastric cancer (AGC) remains uncertain given the lack of 5-year follow-up results.

**Objective:**

To compare the 5-year follow-up results in patients with clinically AGC enrolled in the Korean Laparoendoscopic Gastrointestinal Surgery Study (KLASS)-02 randomized clinical trial who underwent laparoscopic or open distal gastrectomy.

**Design, Setting, and Participants:**

The KLASS-02, a multicenter randomized clinical trial, showed that laparoscopic surgery was noninferior to open surgery for patients with locally AGC. The present study assessed the 5-year follow-up results, including 5-year overall survival (OS) and relapse-free survival (RFS) rates and long-term complications, in patients enrolled in KLASS-02. From November 21, 2011, to April 29, 2015, patients aged 20 to 80 years diagnosed preoperatively with locally AGC were enrolled. Final follow-up was on June 15, 2021. Data were analyzed June 24 to September 9, 2021.

**Interventions:**

Patients were treated with R0 resection either by laparoscopic gastrectomy or open gastrectomy as the full analysis set of the KLASS-02 trial.

**Main Outcomes and Measures:**

Five-year OS and RFS rates, recurrence patterns, and long-term surgical complications were evaluated.

**Results:**

This study enrolled a total of 1050 patients. A total of 974 patients were treated with R0 resection; 492 (50.5%) in the laparoscopic gastrectomy group (mean [SD] age, 59.8 [11.0] years; 351 men [71.3%]) and 482 (49.5%) in the open gastrectomy group (mean [SD] age, 59.4 [11.5] years; 335 men [69.5%]). In patients who underwent laparoscopic and open distal gastrectomy, the 5-year OS (88.9% vs 88.7%) and RFS (79.5% vs 81.1%) rates did not differ significantly. The most common types of recurrence were peritoneal carcinomatosis (73 of 173 [42.1%]), hematogenous metastases (36 of 173 [20.8%]), and locoregional recurrence (23 of 173 [13.2%]), with no between-group differences in types of recurrence at each cancer stage. The correlation between 3-year RFS and 5-year OS at the individual level was highest in patients with stage III gastric cancer (ρ = 0.720). The late complication rate was significantly lower in the laparoscopic than in the open surgery group (32 of 492 [6.5%] vs 53 of 482 [11.0%]). The most common type of complication in both groups was intestinal obstruction (13 of 492 [2.6%] vs 24 of 482 [5.0%]).

**Conclusions and Relevance:**

The 5-year outcomes of the KLASS-02 trial support the 3-year results, which is the noninferiority of laparoscopic surgery compared with open gastrectomy for locally AGC. The laparoscopic approach can be recommended in patients with locally AGC to achieve the benefit of low incidence of late complications.

**Trial Registration:**

ClinicalTrials.gov Identifier: NCT01456598

## Key Points

**Question:**

What is the oncologic safety profile of laparoscopic distal gastrectomy for the treatment of clinically advanced gastric cancer in terms of 5-year survival?

**Findings:**

In this randomized clinical trial of 1050 patients, in patients who underwent laparoscopic or open distal gastrectomy, the 5-year overall survival rates (88.9% vs 88.7%) and relapse-free survival rates (79.5% vs 81.1%) did not differ significantly. The late complication rate was significantly lower in the laparoscopic group than in the open group (6.5% vs 11.0%).

**Meaning:**

The 5-year follow-up results of the Korean Laparoendoscopic Gastrointestinal Surgery Study (KLASS)-02 trial support the rationale for laparoscopic surgery in patients with locally advanced gastric cancer.

## Teaser

This randomized clinical trial investigates the oncologic safety profile of laparoscopic distal gastrectomy compared with open gastrectomy for the treatment of clinically advanced gastric cancer.

## Introduction

The second multicenter randomized clinical trial (RCT) of the Korean Laparoscopic Gastrointestinal Surgery Study Group (KLASS-02) was launched in response to oncologic concerns about the technical appropriateness of laparoscopic D2 lymphadenectomy for locally advanced gastric cancer (AGC).^1^ An independent quality control study (KLASS-02-QC) was performed to qualify the participating surgeons before the RCT.^2,3^ The KLASS-02 RCT concluded that laparoscopic distal gastrectomy performed by qualified surgeons was noninferior in oncologic outcomes to open surgery for locally AGCs.^4^ Because its primary end point was 3-year relapse-free survival (RFS), the length of the trial may have been insufficient to determine the relative long-term outcomes of laparoscopic surgery, as some recurrences are diagnosed more than 3 years after surgery. In addition, the event rate in the KLASS-02 RCT was lower than expected as a high proportion of pathologic early GCs showed low relapse rates. Therefore, patients enrolled in this trial should be followed up for longer than 3 years to determine the clinical efficacy and safety of laparoscopic surgery for locally AGCs.

Traditionally, the 5-year overall survival (OS) rate has been the parameter for determining improved outcomes of experimental treatments for GC in RCTs. This end point can be easily measured and interpreted, although it requires long-term observation and is therefore costly. The effectiveness and safety of curative resection for solid cancers can be assessed more rapidly and efficiently in RCTs by measuring 3-year RFS or disease-free survival rate.^5,6,7^ A meta-analysis of adjuvant RCTs for stage II or III GCs also revealed that 3-year RFS rate may be a surrogate measure of OS.^8^ However, the relevance of 3-year RFS rate as a primary end point replacing 5-year OS rate in patients with clinical stage II or III GCs, including patients with overestimated stages, has not, to our knowledge, been determined. In addition, recently introduced treatment regimens, including chemotherapeutic and targeting agents and checkpoint inhibitors, may improve the survival of patients with GC who experience recurrence.^9,10,11^ Therefore, the oncologic efficacy of laparoscopic surgery for locally AGCs may be better determined by evaluating 5-year follow-up results in patients enrolled in the KLASS-02 RCT.

The KLASS-02 RCT also reported that laparoscopic surgery reduced the rate of late complications compared with open surgery.^4^ In particular, the rate of intestinal obstruction was significantly lower in the laparoscopic group than in the open surgery group. However, population-based and cohort studies have shown that a significant proportion of patients experience intestinal obstruction 3 or more years after abdominal surgery.^12,13^ Therefore, the benefits of laparoscopic surgery, including long-term safety outcomes, should be evaluated 3 or more years after surgery. The aim of the present study was to compare the 5-year follow-up results, including 5-year OS and RFS rates and long-term complications, in patients enrolled in the KLASS-02 RCT.

## Methods

### Study Design and Participants

The KLASS-02 RCT was an investigator-initiated, randomized, controlled, parallel-group, and noninferiority trial comparing laparoscopic D2 lymphadenectomy with conventional open surgery in patients with locally AGCs. The study protocol, surgical quality control, short-term outcomes, and primary end point of this trial have been previously reported (Supplement 1).^1,4,14^

This trial was conducted in accordance with the World Medical Association Declaration of Helsinki. The protocol for data collection was approved by the institutional review board of all participating hospitals. All data were collected via a web-based database system and monitored by an independent committee organized by the clinical trial center of Ajou University Hospital. All patients provided written informed consent. This study followed the Consolidated Standards of Reporting Trials (CONSORT) reporting guidelines.

### Definitions

OS was defined as the time from surgery to the date of last follow-up or death from any cause. RFS was defined as the time from surgery to recurrence or death for any reason. Locoregional recurrence was defined as any clinically proven tumor relapse within the remnant stomach, anastomosis site, or regional lymph nodes at the site of surgery. Hematogenous recurrence was defined as any clinically proven tumor relapse at distal organs outside the operated site, such as the liver, lungs, brain, adrenal glands, and skin. Distant lymph node metastasis was defined as any tumor relapse at lymph nodes, including para-aortic and retroperitoneal lymph nodes. Long-term complications were defined as complications appearing more than 21 days after surgery and could be associated with in-hospital care.

### Randomization and Masking

Patients were randomly assigned (1:1) to undergo laparoscopic or open surgery. A randomized block design was implemented for stratification randomization, with each investigator as the stratification factor to reduce the bias caused by technical differences among surgeons. The investigators were masked to randomization sequence, with the random assignment performed at the coordinating center. However, neither surgeons nor patients were masked to treatment assignment.

### Procedures

Patients in both groups underwent distal gastrectomy with D2 lymphadenectomy and total omentectomy. Reconstruction methods included Billroth I, Billroth II, and Roux-en-Y gastrojejunostomy, with the method in each patient depending on tumor location and/or surgeon preference.^4^ Adjuvant chemotherapy was recommended for all patients with pathological stage II or greater. Chemotherapy regimens included the following: (1) combination tegafur, gimeracil, and oteracil (TS-1) monotherapy or (2) oxaliplatin plus capecitabine (Xeloda).

All patients were postoperatively assessed every 3 months during the first 2 years and then every 6 months for the next 3 years. Abdominopelvic computed tomography (CT) was mandatory every 6 months for the first 3 years and every 6 months or annually thereafter, and gastroscopy was scheduled 1 year from the date of surgery. Recurrence was defined as occurring only if (1) it was radiologically confirmed by abdominopelvic CT, whole-body positron emission tomography CT, magnetic resonance imaging of the liver, or bone scan; (2) it was confirmed endoscopically or by excisional biopsy for locoregional recurrence; or (3) peritoneal carcinomatosis or distant lymph-node involvement was confirmed by laparoscopic exploration, relaparotomy, or ultrasonography-guided biopsy.

### Statistical Analysis

The KLASS-02 RCT trial hypothesized that the 3-year RFS rate in the open surgery group would be 72%, with a hazard ratio (HR) of 1.43 set as the noninferiority margin, corresponding to an 8% 3-year RFS rate margin.^15^ Based on a 1-sided type I error of 2.5%, a dropout rate of 10%, and evaluation using a log-rank test, 1050 patients (525 per group) were calculated as needed to achieve a power of 90%.

The full analysis set (FAS) data were analyzed using R statistics, version 4.1 (R Foundation), and SPSS statistics, version 25 (IBM Corp). Differences in proportions were analyzed using the χ^2^ or Fisher exact tests, and differences in distributions were analyzed using *t* test or the Mann-Whitney *U* test. A 2-sided *P* value < .05 was considered statistically significant. The Jonckheere trend test was used to determine differences in the distribution of recurrences. Survival, recurrence, and late complication rates in the 2 groups were determined using the Kaplan-Meier method, with differences determined by log-rank tests. The individual-level associations between the probabilities of 3-year RFS and 5-year OS were determined by Spearman rank correlation analysis.^16,17^ Data were analyzed June 24 to September 9, 2021.

## Results

### Patients

Of the 1050 patients enrolled between November 21, 2011, and April 29, 2015, 76 were excluded because of withdrawal of consent, noncurative treatment, operative mortality, or loss to follow-up (eFigure 1 in Supplement 2). Six patients in the laparoscopic group and 11 in the open group were crossed over in the FAS data set, with these patients reassigned to the opposite surgery group based on treatment intent. Thus, 5-year outcomes were analyzed in 492 patients in the laparoscopic group (mean [SD] age, 59.8 [11.0] years; 351 men [71.3%]; 141 women [28.7%]) and 482 in the open group (mean [SD] age, 59.4 [11.5] years; 335 men [69.5%]; 147 women [30.5%]). A total of 974 patients were treated with R0 resection. The baseline clinicopathological characteristics, including the extent of gastrectomy or lymphadenectomy, the number of retrieved lymph nodes, TNM stages, and completion of adjuvant chemotherapy, did not differ significantly in these 2 groups (Table 1).

**Table 1.** Patient Clinicopathological Characteristics

Variable	No. (%)	
	Laparoscopy (n = 492)	Open (n = 482)
Age, mean (SD), y	59.8 (11.0)	59.4 (11.5)
Sex		
Men	351 (71.3)	335 (69.5)
Women	141 (28.7)	147 (30.5)
BMI, mean (SD)^a^	23.5 (2.9)	23.7 (3.3)
ASA group		
I	239 (48.6)	235 (48.8)
II	228 (46.3)	225 (46.7)
III	25 (5.1)	22 (4.6)
Extent of resection		
Distal gastrectomy	477 (97.0)	470 (97.5)
Total gastrectomy	15 (3.0)	12 (2.5)
Extent of lymphadenectomy		
<D2	0 (0)	3 (0.6)
D2	492 (100.0)	479 (99.4)
Tumor size, mean (SD), cm	4.6 (2.5)	4.6 (2.3)
Lymph nodes, mean (SD)		
Retrieved	46.8 (18.1)	47.2 (16.2)
Metastatic	3.6 (6.1)	3.4 (5.7)
Histology		
Differentiated	197 (40.0)	187 (38.8)
Undifferentiated	286 (58.1)	278 (57.7)
Others	9 (1.8)	17 (3.5)
Pathological T classification		
T1	137 (27.8)	125 (25.9)
T2	104 (21.1)	113 (23.4)
T3	132 (26.8)	135 (28.0)
T4	119 (24.2)	109 (22.6)
Pathological N classification		
N0	223 (45.3)	219 (45.4)
N+	269 (54.7)	263 (54.6)
Pathological 8th TNM stage		
I	178 (36.2)	165 (34.2)
II	148 (30.1)	167 (34.6)
III	166 (33.7)	150 (31.1)
IV	0 (0)	0 (0)
Neoadjuvant chemotherapy	0 (0)	0 (0)
Postoperative adjuvant chemotherapy		
Received	298 (60.6)	299 (62.0)
Tegafur/gimeracil/oteracil (TS-1)	161 (54.0)	185 (61.9)
Capecitabine (Xeloda) + oxaliplatin	100 (33.6)	81 (27.1)
Fluorouridine	19 (6.4)	15 (5.0)
Other	18 (6.0)	18 (6.0)
Completed	213 (76.1)	212 (75.4)
Dose reduction	100 (33.6)	102 (34.1)
Time interval to adjuvant chemotherapy, mean (SD), weeks	5.0 (2.0)	5.1 (1.7)

Abbreviations: ASA, American Society of Anesthesiologists; BMI, body mass index.

^a^ Calculated as weight in kilograms divided by height in meters squared.

### Overall and Relapse-Free Survival

**Figure 1.**
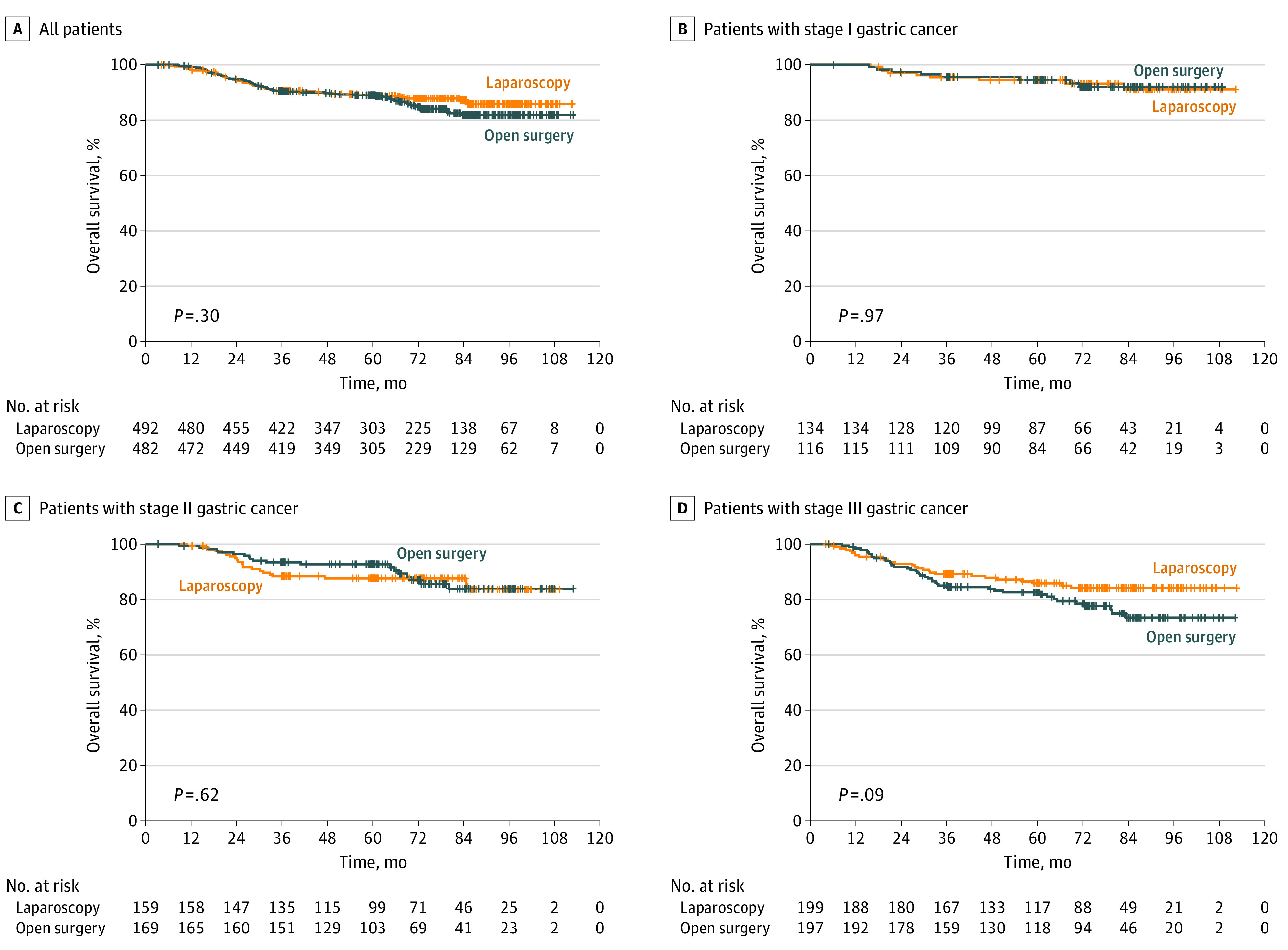
Kaplan-Meier Analyses of Overall Survival After Laparoscopic Gastrectomy and Open Gastrectomy Overall survival in all patients (A) and patients with stage I (B), stage II (C), and stage III (D) gastric cancer, based on the 8th TNM staging system (the full analysis set data set).

The last enrolled patient was monitored for at least 5 years. The overall median (IQR) follow-up time was 69.4 (3.3-112.9) months, 68.0 months in the laparoscopy group and 70.1 months in the open group. The 5-year OS rates in the FAS data set did not differ significantly in patients who underwent laparoscopic (88.9%; 95% CI, 86.0%-91.8%) and open (88.7%; 95% CI, 85.8%-91.6%) distal gastrectomy (Figure 1A). Similarly, 5-year RFS rates were similar in patients who underwent laparoscopic (79.5%; 95% CI, 75.9%-83.2%) and open (81.1%; 95% CI, 77.7%-84.8%) gastrectomy (eFigure 2A in Supplement 2). Subanalyses according to pathologic stages are shown in eFigures 3 and 4 in Supplement 2. The patients’ characteristics and OS values in the intention-to-treat (ITT) data set are shown in eTable 1 and eFigures 5 and 6 in Supplement 2. The OS values of the patients who completed adjuvant chemotherapy are shown in eFigure 7 in Supplement 2.

The number of deaths or recurrences was 108 (21.9%) in the laparoscopy group and 101 (20.9%) in the open group. A total of 58 patients (11.8%) in the laparoscopy group and 69 patients (14.3%) in the open group died during the follow-up period, and 93 (18.9%) and 80 (16.6%), respectively, experienced recurrences. The most common type of recurrence was peritoneal carcinomatosis (73 of 173 [42.1%]), followed by hematogenous metastases (36 of 173 [20.8%]), locoregional recurrence (23 of 173 [13.2%]), and distal lymph node metastases (17 of 173 [9.8%]). There were no between-group differences in locations of recurrence (eFigure 8 in Supplement 2). Table 2 lists a detailed distribution of recurrence according to tumor stage and number of postoperative years in the 2 groups. More than 80% of all recurrences (144 of 173) observed during the follow-up period were recorded within the first 3 postoperative years. The number of recurrences after 3 postoperative years tended to be higher in the laparoscopic group, but the difference between the 2 groups was not statistically significant.

**Table 2.** Recurrence Patterns During Each Postoperative Year

Pathologic stage/recurrence patterns	No. of recurrences (stage I/II/III)	Recurrences in each postoperative year, No.							
		1 y	2 y	3 y	4 y	5 y	6 y	7 y	>7 y
Laparoscopy (n = 492)	93	26	34	13	8	5	1	2	4
Locoregional	1/5/5	0/0/1	0/1/0	0/2/1	0/1/0	0/0/2	1/0/0	0/1/0	0/0/1
Hematogenous	3/5/11	1/3/5	1/2/3	0/0/0	1/0/1	0/0/1	0/0/0	0/0/0	0/0/1
Peritoneal	1/6/31	0/2/8	0/3/11	1/1/4	0/0/4	0/0/2	0/0/0	0/0/0	0/0/2
Distant LN	0/3/8	0/1/3	0/1/3	0/1/1	0/0/0	0/0/0	0/0/0	0/0/1	0/0/0
Mixed	0/5/9	0/1/1	0/3/6	0/1/1	0/0/1	0/0/0	0/0/0	0/0/0	0/0/0
Open (n = 482)	80	21	31	19	5	2	2	0	0
Locoregional	1/6/5	0/2/1	0/4/2	0/0/1	0/0/0	0/0/1	1/0/0	0/0/0	0/0/0
Hematogenous	2/2/13	1/1/6	0/1/3	0/0/3	1/0/0	0/0/0	0/0/1	0/0/0	0/0/0
Peritoneal	0/6/29	0/0/5	0/0/12	0/4/10	0/1/2	0/1/0	0/0/0	0/0/0	0/0/0
Distant LN	0/0/6	0/0/2	0/0/4	0/0/0	0/0/0	0/0/0	0/0/0	0/0/0	0/0/0
Mixed	1/2/7	1/0/2	0/1/4	0/1/0	0/0/1	0/0/0	0/0/0	0/0/0	0/0/0

Abbreviation: LN, lymph node.

Median (IQR) survival times from recurrence to death were 435 (97-435) days in patients with hematogenous metastases, 287 (142-413) days in patients with peritoneal carcinomatosis, 255 (184-371) days in patients with distant lymph node metastases, and 179 (40-344) days in patients with locoregional recurrences. The overall correlation between 3-year RFS and 5-year OS on an individual level for all patients was 0.447 (95% CI, 0.393-0.498) for all patients. Subgroup analysis showed that ρ values for patients with stages I, II, and III GCs were 0.242, 0.469, and 0.720, respectively (Table 3).

**Table 3.** Correlations Between 3-Year Relapse-Free Survival and 5-Year Overall Survival at the Individual Level

Pathologic stage, No.	ρ (95% CI)^a^
Full analysis set (n = 974)	0.447 (0.393-0.498)
I (n = 343)	0.242 (0.138-0.341)
II (n = 315)	0.469 (0.373-0.555)
III (n = 316)	0.720 (0.655-0.775)

^a^ The ρ represents Spearman rank correlation coefficient between overall survival and relapse-free survival.

### Long-term Surgical Complications

**Figure 2.**
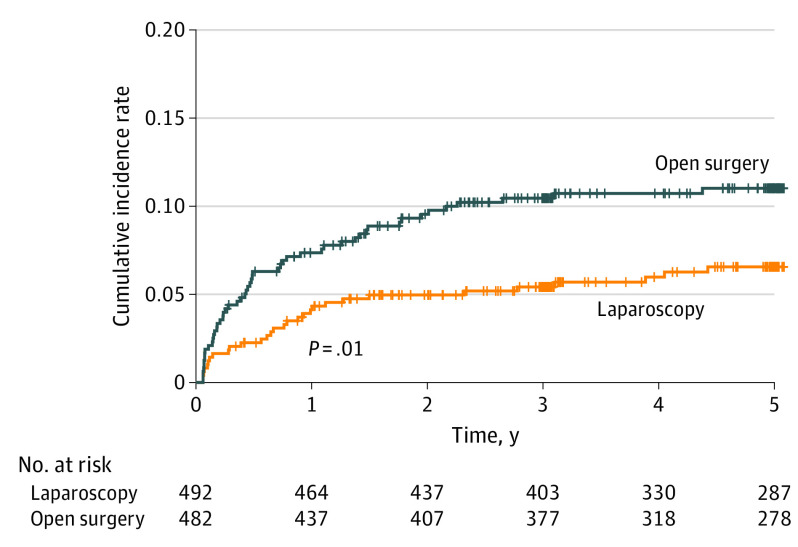
Cumulative Incidence Rates of Patients at Risk of Late Complications Over Time After Laparoscopic Gastrectomy and Open Gastrectomy

Complications rates in the laparoscopy group were significantly lower in the laparoscopic group than in the open group (32 of 492 [6.5%] vs 53 of 482 [11.0%]; *P* = .01) (eTable 2 in Supplement 2). Figure 2 shows the cumulative incidence curves in the 2 groups over 5 postoperative years. The cumulative rate of late complications was significantly lower in the laparoscopy group than in the open group. Intestinal obstruction was the most common surgical complication (37 of 85 [43.5%]), with rates of intestinal obstruction (13 of 492 [2.6%] vs 24 of 482 [5.0%]; *P* = .06) and chronic wound complications (3 of 492 [0.6%] vs 9 of 482 [1.9%]; *P* = .08) tending to be lower in the laparoscopy group than in the open group. Major complications were more frequent in the open group, although the difference was not statistically significant.

## Discussion

The KLASS-02 RCT study showed that laparoscopy was noninferior to open surgery, as determined by 3-year RFS rates, and laparoscopic surgery was associated with a lower rate of complications in patients with locally AGCs.^4^ The 5-year follow-up results in this trial also showed no significant differences in RFS and OS rates between the laparoscopic and open surgery groups. In addition, the lower rate of late complications in the laparoscopy group indicates that laparoscopic gastrectomy provides substantially better surgical outcomes than open surgery. These results indicate that laparoscopic surgery is a clinically relevant procedure for patients with locally AGCs, as shown by long-term oncologic and surgical outcomes.

The present study evaluated the survival of patients enrolled in the KLASS-02 RCT after the planned 3-year trial length because a significant proportion of recurrences in patients with locally AGCs experience recurrences more than 3 years after surgery.^18,19^ For example, a retrospective study of patients with AGC treated with curative resection and adjuvant chemotherapy showed that approximately 20% to 30% of peritoneal or hematogenous recurrences were diagnosed 4 to 5 years after surgery. By contrast, most lymph node recurrences occurred within the first 3 postoperative years.^18^ Because major sites of recurrence after curative resection include the peritoneum and liver, the rate of recurrence (the primary end point of the KLASS-02 RCT) should be analyzed for at least 5 years after surgery to determine the noninferiority of laparoscopic surgery to open surgery in patients with locally AGCs. Although the number of recurrent events after 3 years was 76 in the laparoscopic group and 72 in the open group,^4^ the current study found that recurrences were diagnosed in 20 patients (21.5% of total recurrences) in the laparoscopic group and in 9 (11.3% of total recurrences) in the open group 4 to 5 years after surgery. Despite these apparent differences, 5-year RFS rates did not differ significantly in the 2 groups. These findings indicate that the similar survival outcomes after laparoscopic and open surgeries for locally AGCs were substantially maintained for at least 5 years after surgery.

Previous prospective RCTs, including the KLASS-02 RCT, have provided evidence for the oncologic safety of laparoscopic surgery for local AGCs.^4,20,21^ These trials reported outcomes for only 3 years, the first KLASS trial (KLASS-01) RCT comparing 2 procedures in patients with clinical early GCs reported 5-year or greater follow-up results.^22^ In that trial, 477 patients had been diagnosed with pathologic AGCs, with a post hoc subgroup analysis finding no difference in OS rates between the laparoscopic and open surgery groups (HR, 0.71 vs 0.81).^22^ However, the subgroup analysis may have been insufficiently powered to determine the oncologic feasibility of laparoscopic surgery for AGCs. Recent meta-analyses, however, have indicated that laparoscopic surgery for locally AGCs is oncologically feasible.^23^ Most retrospective studies included in that meta-analysis had a limited follow-up period of 50 months or less. Only 1 propensity-matched study followed up patients for a longer period (range, 88-100 months), but found no difference in survival between the laparoscopic and open surgery groups.^24^ The current study reports 5-year follow-up results of a well-designed prospective study comparing laparoscopic and open procedures for patients with locally AGCs.

The choice of 3-year RFS rate as the primary end point of the KLASS-02 RCT was based on results of previous large-scale RCTs in patients with locally AGCs.^25,26^ Determining the 5-year OS rate, a more traditional end point in oncologic RCTs, requires an extended follow-up period and is more costly. Moreover, the oncologic outcomes of surgical procedures can be altered by other causes of death or treatments for recurrent disease. Three-year RFS rates are considered a reasonable surrogate for 5-year OS rates in the RCTs of patients with various solid tumors who undergo curative resection.^5,6,7,8^ One meta-analysis showed that the 3-year RFS rate correlated significantly with the 5-year OS rate in RCTs of patients receiving adjuvant chemotherapy for locally AGCs. However, the present study showed a lower correlation between 3-year RFS and 5-year OS rates. This may have been attributable to the inclusion in the KLASS-02 RCT of a significant proportion of patients with early-stage GC, who have a low recurrence rate, as KLASS-02 enrolled patients according to clinical stage. In addition, recent advanced treatment modalities for patients with recurrent GC can increase the time from recurrence to death, resulting in a lower correlation between 3-year RFS and 5-year OS rates. Because clinical trials measuring the efficacy of surgical procedures assess recurrences after resection, 3-year RFS rate may be an attractive primary end point. Measurement of the precise time of recurrence would be a prerequisite for using 3-year RFS rate as the primary end point, as both KLASS-02 and the present study provided detailed information, including the sites of all recurrent events. Subgroup analysis according to pathologic stage found that the 3-year RFS rate of patients with stage III GC correlated with 5-year OS rate (ρ = 0.720), suggesting that 3-year RFS rate may be a good end point in patients with pathologic stage III GC. However, 3-year RFS rate may not replace 5-year OS rate as the primary end point for patients with stages I and II GC.

The low incidence of late complications was one of the advantages of laparoscopic surgery reported in the KLASS-02 RCT. Most other long-term complications showed little increase after 3 years, whereas newly developed intestinal obstructions were observed in 5 patients in the laparoscopic group and 9 in the open group after the end of 3 years. Large-scale retrospective data with long-term follow-up revealed that the mean interval to reoperation owing to intestinal obstruction after GC surgery was approximately 2 years, suggesting that a significant proportion of patients with symptomatic intestinal obstructions could be diagnosed within 3 years after surgery.^27^ The present study showed that, after 3 years, the incidence of intestinal obstruction increased 33.3% in the laparoscopic group and 30.0% in the open group, with the difference of incidence between the 2 groups maintained after 3 years. However, a recent RCT reported that an antiadhesive agent could significantly reduce the incidence of intestinal obstruction after open gastrectomy for GC without increasing adverse events.^28^ By contrast, the barbed-suture materials frequently used in laparoscopic surgery could lead to the development of postoperative obstruction.^29^ Further studies are needed to assess the benefits of laparoscopic surgery for GCs, including the lower incidence of intestinal obstruction.

### Limitations

The present study showed that laparoscopic surgery maintained substantial oncologic and surgical outcomes during a 5-year follow-up. However, the study has some limitations, which included unconditionally performing laparoscopic surgery in all patients with clinically AGC. First, the KLASS-02 RCT enrolled patients with GC with metastasis in the perigastric lymph nodes or lymph nodes around the left gastric artery by clinical staging. The present study could not determine the oncologic safety of laparoscopic surgery in patients with far-advanced stage GC, who were underestimated in preoperative evaluations. A Japanese multicenter cohort study in patients with Borrmann type 4 AGC found that long-term oncologic outcomes were better after open surgery than after laparoscopic surgery.^30^ Moreover, a recent large-scale retrospective analysis in patients with GC with serosa-exposed and large-sized tumors found that 5-year survival outcomes were better after open surgery than after laparoscopic surgery, although the 3-year outcomes did not differ in these 2 groups.^31^ Although the current study did not report statistically significant differences, peritoneal recurrence after 3 years was reported in only 2 patients with stage III tumors after open surgery, compared with 8 after laparoscopic surgery. Taken together, these results suggest caution in performing laparoscopic surgery in patients with far-advanced GC. The second limitation was that the advantage of laparoscopic surgery in terms of long-term complications was not confirmative. The surgeries for intestinal obstruction could be performed in other hospitals owing to the urgent condition of the patients. Thus, some long-term complications may have been omitted. In addition, wound complications, such as ventral hernia, would be diagnosed by postoperative year 5. Therefore, long-term follow-up data after 5 years for those patients would be required.

## Conclusions

In conclusion, the oncologic and surgical outcomes of the 5-year follow-up of the KLASS-02 RCT supported noninferiority of laparoscopic surgery for locally AGCs compared with open surgery, which were found in the previous 3-year end points of this trial. Considering the low complication rate of laparoscopic surgery, we suggest that the laparoscopic approach for patients with locally AGCs replace conventional open surgery.

## Notes

Supplement 1. Trial Protocol

Supplement 2. **eFigure 1. **Trial Profile **eFigure 2. **Kaplan-Meier Analyses of Relapse-Free Survival Following Laparoscopic Gastrectomy and Open Gastrectomy (cStage FAS Data Set) **eFigure 3. **Kaplan-Meier Analyses of Overall Survival Following Laparoscopic Gastrectomy and Open Gastrectomy (pStage FAS Data Set) **eFigure 4. **Kaplan-Meier Analyses of Relapse-Free Survival Following Laparoscopic Gastrectomy and Open Gastrectomy (pStage FAS Data Set) **eFigure 5. **Kaplan-Meier Analyses of Overall Survival Following Laparoscopic Gastrectomy and Open Gastrectomy (cStage ITT Data Set) **eFigure 6. **Kaplan-Meier Analyses of Overall Survival Following Laparoscopic Gastrectomy and Open Gastrectomy (pStage ITT Data Set) **eFigure 7. **Kaplan-Meier Analyses of Overall Survival Following Laparoscopic Gastrectomy and Open Gastrectomy in Patients Who Completed Adjuvant Chemotherapy (pStage FAS Data Set) **eFigure 8. **Recurrence Patterns in Patients Who Underwent Laparoscopic Gastrectomy and Open Gastrectomy **eTable 1. **Patient Clinicopathological Characteristics **eTable 2. **Distribution of Long-term Surgical Complications (>21 Postoperative Days) in Patients Who Underwent Laparoscopic Gastrectomy and Open Gastrectomy

Supplement 3. Nonauthor Collaborators

Supplement 4. Data Sharing Statement
