# Supplementary material for: Laparoscopic vs Open Distal Gastrectomy for Locally Advanced Gastric Cancer: 5-Year Outcomes of the KLASS-02 Randomized Clinical Trial
Source: JAMA Surg. 2022 Jul 20;157(10):879–86. doi: 10.1001/jamasurg.2022.2749 (PMC9301593; doi:10.1001/jamasurg.2022.2749)
Supplement: Supplement 1. — Trial Protocol [file jamasurg-e222749-s001.pdf]

# Clinical Study Protocol

Prospective Multicenter Randomized Controlled Clinical Trial for Comparison between Laparoscopic and Open Subtotal Gastrectomy with D2 Lymph Node Dissection for Locally Advanced Gastric Cancer

Protocol No.: KLASS-02
Version: 5.2

Approval of the first version: Dec 09, 2010
Approval of the last update version: April 05, 2018

Study site: Ajou University Hospital
Principal Investigator: Prof. Sang-Uk Han
Department of Surgery, Ajou University Hospital

# TABLE OF CONTENTS

1. Study title ... 5
2. Participating sites ... 5
3. Principal Investigator and Sub-Investigator ... 6
4. Study objectives ... 6
4.1 Primary objective ... 6
4.2 Secondary objective ... 6
5. Background ... 6
5.1 Feasibility of laparoscopic gastric surgery ... 6
5.2 Treatment options for advanced gastric cancer ... 8
5.3 Rationale for D2 lymphadenectomy in gastric cancer surgery ... 9
5.4 Background to the need for this clinical trial ... 9
6. Ethical considerations ... 10
7. Surgical procedures used in the study ... 11
8. Study duration ... 11
9. Selection criteria and sample size calculation ... 11
9.1 Inclusion criteria ... 11
9.2 Exclusion criteria ... 11
9.3 Target patient population and sample size determination ... 12
10. Study design ... 13
10.1 Qualification of participating surgeons and standardization and quality control of surgery 13
10.2 Management of participating sites and patient enrolment ... 13
10.3 Informed consent process ... 14
10.4 Treatment plan for enrolled patients ... 15
10.5 Postoperative patient management ... 17
10.6 Postoperative care depending on pathological outcomes ... 17
10.7 Efficacy and safety assessment ... 18
11. Assessment items, clinical exam items, and observational assessment method ... 18
11.1 Baseline assessment items ... 18
11.2 Assessment items on the operation day ... 20
11.3 Assessment items during the postoperative hospital stay ... 21

11.4 Assessment items on postoperative day 25 ........ 22
11.5 Assessment items at postoperative months 3, 6, 9, 12, 15, 18, 21, 24, 27, 30, 33, 36 ........ 22
11.6 Assessment items related to late complications ........ 23
11.7 Relapse-related assessment items ........ 24
11.8 Survival- and death-related assessment items ........ 25
11.9 Assessment items related to adjuvant chemotherapy ........ 25
11.10 Study flow shhet ........ 25
12. Criteria for the continuation of the study ........ 27
12.1 Withdrawal criteria ........ 27
12.2 Conversion from laparoscopic distal subtotal gastrectomy to total gastrectomy ........ 27
12.3. Conversion from distal subtotal gastrectomy to total gastrectomy ........ 27
12.4 Cases requiring combined resection of adjacent organs for radical resection ........ 27
13. Termination of the study ........ 28
14. Subject safety protection and appropriate responses to adverse events ........ 28
15. Analysis set and statistical analysis plan ........ 28
15.1 Analysis sets ........ 28
15.2 Statistical methods ........ 29
15.3 Analysis time points and the final analysis ........ 31
16. Efficacy assessment criteria and assessment methodology ........ 31
16.1 Efficacy determination ........ 31
16.2 Evaluation of the efficacy endpoints ........ 31
17. Criteria for safety assessment and methods of assessment and reporting ........ 33
17.1 Methods of postoperative complication assessment and reporting ........ 33
17.2 Safety evaluation of laparoscopic surgery based on early postoperative complications ........ 35
17.3 Postoperative 90-day mortality and its reporting ........ 36
17.4 Evaluation of adverse events in patients under adjuvant chemotherapy ........ 37
18. Adverse event reporting ........ 37
18.1 Serious adverse events ........ 37
18.2 Reporting of serious adverse events ........ 37
19 Governing bodies of the study and their roles ........ 38
19.1 Steering Committee ........ 38
19.2 Data and Safety Monitoring Committee ........ 38

19.3 Advisory Committee ..... 39
19.4 Clinical Research Organization ..... 39
20. Study outcome database management ..... 39
20.1 Study site monitoring ..... 39
20.2 Case report and database management ..... 40
21. Subject information sheet and informed consent form ..... 40
22. Additional use of data ..... 41
23. Study-related data and document archiving ..... 41
23.1 Data management ..... 41
23.2 Confidentiality ..... 42
23.3 Access to the study-related data and literature ..... 42
23.4 Quality control (QC) and quality assurance (QA) of study data ..... 42
24. References ..... 44

## 1. Study title

Full study title: Prospective Multicenter Randomized Controlled Clinical Trial for Comparison between Open and Laparoscopic Subtotal Gastrectomy with D2 Lymph Node Dissection for Locally Advanced Gastric Cancer

Short title: Open vs. laparoscopic subtotal gastrectomy for locally advanced gastric cancer.

Study code: KLASS-02
(KLASS: Korean Laparoscopy Gastrointestinal Surgery Study Group)

## 2. Participating sites

This clinical trial will be co-conducted at the medical institutions where the surgeons work, whose participation in the present clinical trial has been approved by the ad hoc Review Committee that reviewed the precedence study, KLASS-02 Efficacy of Laparoscopic Subtotal Gastrectomy with D2 Lymphadenectomy for Locally Advanced Gastric Cancer (KLASS-02-RCT) and the Standardization of D2 Lymphadenectomy and Surgical Quality Control (KLASS-02-QC) study. The list of the participating sites may be extended as additional hospitals join the study.

| Study site | Address | PI | Sub-I |
|---|---|---|---|
| Ajou University Hospital | San-5 Woncheon-dong, Yeongtong-gu, Suwon, Gyeonggi-do | Sang-Uk Han | Hoon Hur |
| Severance Hospital, Yonsei University Health System | 250 Seongsan-ro (134 Sinchon-dong), Seodaemun-gu, Seoul | Woo Jin Hyung | Hyoung-Il Kim<br>Ji Young An |
| Seoul National University Hospital | 101 Daehak-ro, Jongno-gu, Seoul | Han-Kwang Yang | Hyuk-Joon Lee, Seong-Ho Kong |
| Dong-A University Hospital | 3-1 Dongdaesin-dong, Seo-gu, Busan | Min-Chan Kim | |
| Yeouido St. Mary's Hospital, Catholic University of Korea | 62 Yeouido-dong, Yeongdeungpo-gu, Seoul | Wook Kim | |
| Incheon St. Mary's Hospital, Catholic University of Korea | 56 Dongsu-ro, Bupyeong-gu, Incheon | Jin-Jo Kim | |
| Chonnam National University Hwasun Hospital | 106 Ilsam-ri Hwasun-eup, Hwasun-gun, Jeollanam-do | Young Kyu Park | Seong Yeop Ryu |
| Keimyung University Dongsan Medical Center | 56 Dalseong-ro, Jung-gu, Daegu | Seung Wan Ryu | |
| Seoul National University Bundang Hospital | 300 Gumi-dong, Bundang-gu, Seongnam-si, Gyeonggi-do | Hyung-Ho Kim | Do Joong Park |
| Chung-Ang University Hospital | 102 Heukseok-ro, Dongjak-gu, Seoul | Jong Won Kim | |
| Soonchunhyang University Bucheon Hospital | 170 Jomaru-ro, Wonmi-gu, Bucheon-si, Gyeonggi-do. | Gyu Seok Cho | |
| Ewha Womans University Mokdong Hospital | 1071 Anyangcheon-ro, Yangcheon-gu, Seoul | Joo-Ho Lee | |
| National Cancer Center | 323 Ilsan-ro, Ilsandong-gu, Goyang-si Gyeonggi-do | Young Woo Kim | Keun Won Ryu |

(PI: principal investigator; Sub-I: sub-investigator)

## 3. Principal Investigator and Sub-Investigator

Coordinating Principal Investigator: Prof. Sang-Uk Han (PI), Ajou University Hospital
Study Monitor: Prof. Hoon Hur (Sub-I), Ajou University Hospital

## 4. Study objectives

### 4.1 Primary objective

The primary objective is to investigate the non-inferiority of laparoscopic subtotal gastrectomy with D2 lymph node dissection compared to open subtotal gastrectomy for the treatment of patients with locally advanced gastric cancer in terms of the 3-year relapse-free survival (RFS) rate.

### 4.2 Secondary objective

The secondary objective is to compare laparoscopic subtotal gastrectomy and D2 lymphadenectomy, and open subtotal gastrectomy using the following variables:

- Postoperative complications
  - Early postoperative complications: onset within 21 days after surgery
  - Late postoperative complications: onset 21 days after surgery
- Postoperative mortality (90-day all-cause mortality after surgery)
- Postoperative surgical recovery profile (postoperative pain, length of hospital stay)
- Postoperative quality of life (scores at postoperative day 25 days and year 1)
- Postoperative 3-year overall survival (OS)

## 5. Background

### 5.1 Feasibility of laparoscopic gastric surgery

The safety and effectiveness of laparoscopic gastrectomy and lymphadenectomy for gastric cancer have been reported in the following retrospective studies and based on clinical considerations reported in the literature:

1) The first laparoscopic surgery for gastric cancer was performed in 1992 by *Ohgami et al*.[1] They resected small lesions (≤25 mm) on the anterior wall using a lesion-lifting method and reported the effectiveness of laparoscopic gastrectomy after following up cases of such laparoscopic surgery for five years.

2) The case of a Billroth I reconstruction after performing laparoscopic subtotal gastrectomy using the method which is still currently in use was first reported by *Kitano et al*. in 1994.[2]

3) The first case of laparoscopy-assisted subtotal gastrectomy in early gastric cancer in Korea was reported in 1996 by *Choi et al*.,[3] and the number of laparoscopic gastric cancer procedures rapidly increased over the next decade to over 3600 cases in 2008 (Fig. 1).

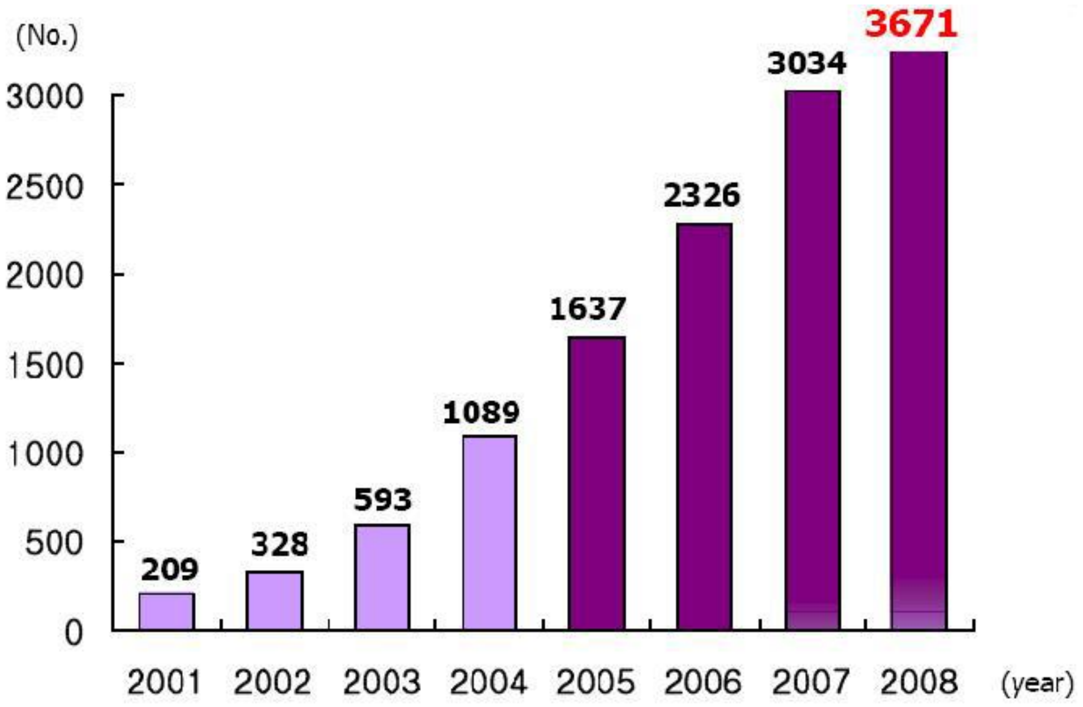


Fig 1. The number of laparoscopic gastric cancer surgical procedures in Korea (from http://www.kgca-i.or.kr)

4) Laparoscopic gastric cancer surgery has been limited to early gastric cancer due to the steep learning curve required for the surgeon to acquire familiarity with laparoscopic surgery and the difficulties associated with extensive lymphadenectomy.

5) Clinical studies on the effectiveness of laparoscopic surgery in patients with early gastric cancer have been continuously reported over the past decades.

6) Korean retrospective studies analyzing the surgical outcomes of 1485 cases of laparoscopic gastric cancer surgery performed in 10 medical institutions since 1998, reported postoperative complication and mortality rates of about 14% and 0.6%, respectively, showing little difference from open surgery.[4,5]

7) *Kitano et al*. reported on the surgical outcomes of 1294 cases of laparoscopic surgery for early gastric cancer performed in multiple centers from 1994 onwards. The 5-year DFS rates were 99.8% for stage IA, 98.7% for stage IB, and 85.7% for stage II, showing no significant difference from the surgical outcomes of open surgery published thus far.[6]

8) Prospective randomized clinical trials (RCT) have also been conducted, albeit with small sample sizes, for the purpose of comparing open and laparoscopic gastric cancer surgery. All these studies reported that laparoscopic surgery had the advantages of reducing intraoperative blood loss and shortening recovery time, with no increase in postoperative complication rate and mortality.[7-10]

Table 1. Clinical Trials comparing laparoscopy and open gastrectomy

| Study | Year of publication, | Country | No of Patients | | LN dissection | Indication |
|---|---|---|---|---|---|---|
| | | | LADG | ODG | | |
| Hayashi et al | 2005, | Japan | 14 | 14 | D1+a | T1a 아 T1b |
| Huscher et al. | 2005, | Italy | 30 | 29 | D1,D2 | All stages |
| Kitano et al | 2002, | Japan | 14 | 14 | D1+a | T1aN0 |
| Lee et al.[10] | 2002, | Korea | 24 | 23 | D2 | T1a or T1b |

9) A prospective randomized clinical study conducted by *Huscher et al*.[8] in gastric cancer patients, including those with advanced gastric cancer that underwent laparoscopy-assisted and open radical subtotal gastrectomy were followed up for five years. No significant difference in 5-year overall and DFS rates were found between the two surgical groups. The drawback of this study was its small sample size (n = 59).

10) In light of the studies conducted thus far as presented above, laparoscopic subtotal gastrectomy and lymphadenectomy for early gastric cancer may be generally accepted. In Korea, there is an ongoing prospective randomized clinical trial (KLASS Trial) with long-term follow-up (from 2006) for early-stage gastric cancer (clinically diagnosed early gastric cancer or advanced gastric cancer without lymph node metastasis or serosal invasion) with the objective of demonstrating the effectiveness of laparoscopic gastric cancer surgery (registered in www.clinicaltrials.gov as NCT00452751) In an interim report, it was concluded that there was no significant difference between the laparoscopic and open gastrectomy groups in terms of surgical complications and postoperative mortality.[11]

**5.2 Treatment options for advanced gastric cancer**

1) The extent of lymphadenectomy in surgery for locally advanced gastric cancer without distant metastasis is still a controversial topic among surgeons.

2) In clinical trials comparing D1 versus D2 lymphadenectomy for advanced gastric cancer conducted in the Western world, D2 lymphadenectomy was reported to increase surgical complications, without showing any increase in survival rates of oncologic significnce.[12-15] However, the validity of these trials have been called into question in view of the surgical skills of the participating surgeons as well as the quality of surgery and pathological examinations.

3) In Eastern countries such as Korea and Japan, however, D2 lymphadenectomy has become a standard lymphadenectomy procedure for advanced gastric cancer as a result of positive evaluations in various clinical studies.[17,18] Furthermore, in clinical trials of D2 versus extended D2 (D2+) lymphadenectomy for locally advanced gastric cancer, it has been demonstrated that D2+ lymph node dissection is not required.[19-22]

4) Supported by these results, gastrectomy including D2 lymphadenectomy and appropriate postoperative adjuvant chemotherapy as indicated by the Japanese Gastric Cancer Association has become a standard procedure for locally advanced gastric cancer without distant metastasis.[23]

Table 2. Randomized control clinical trials for evaluation of difference between D1 and D2 lymphadenectomy

| | | N | Morbidity | | Mortality | | 5 yr survival | |
|---|---|---|---|---|---|---|---|---|
| | | | Rate | p-value | Rate | p-value | Rate | p-value |
| MRC | D1 | 200 | 28.0% | <0.001 | 6.5% | 0.04 | 35% | 0.72 |
| | D2 | 200 | 46.0% | | 13.0% | | 33% | |
| Dutch | D1 | 498 | 25.0% | <0.001 | 4% | 0.04 | 30% | 0.53 |
| | D2 | 498 | 43.0% | | 10% | | 35% | |
| Italian | D1 | 76 | 10.5% | NS | 1.3% | NS | Not evaluated | |
| | D2 | 86 | 16.3% | | 0% | | | |

## 5.3 Rationale for D2 lymphadenectomy in gastric cancer surgery

1) D2 lymphadenectomy is a complicated procedure and has been reported to have higher postoperative complications in clinical trials comparing surgical outcomes of D1 (open surgery) and D2 (laparoscopic surgery) ressections.[13,14]

2) In particular, unlike D1, D2 lymphadenectomy requires dissection of perivascular lymph nodes and those in regions posing difficulty for laparoscopic access.

3) Due to the unconfirmed safety and effectiveness of laparoscopic surgery for D2 lymphadenectomy its application is generally limited to patients with early gastric cancer.

4) To date, cases of laparoscopic D2 lymphadenectomy have been reported only by a few surgeons experienced in laparoscopic surgery, given that it is a challenging procedure requiring a steep learning curve to acquire surgical proficiency, and the surgical outcomes reported were not inferior to open surgery.[24-26]

5) Most surgical outcomes reported to date are based on retrospective analysis. Only a few reports have presented long-term survival rates verifying oncologic safety, and no large-scale prospective study has yet been undertaken.

## 5.4 Background for the need of the present clinical trial

1) Various surgical established procedures such as cholecystectomy and colectomy have indicated that laparoscopic surgery contributes to reducing postoperative pain and the recovery period.

With further development of surgical techniques and instruments, there is also clinical evidence of the effectiveness laparoscopic surgery in addressing malignant tumors.

2) Whereas laparoscopic gastrectomy is now commonly applied to early gastric cancer, spurred by clinical evidence, open surgery is still the standard surgical treatment for advanced gastric cancer given the lack of clinical evidence to support the efficacy of laparoscopic gastrectomy for advanced gastric cancer.

3) In Korea, 38% of patients undergoing gastric cancer surgery are those diagnosed with stages T2–T3 (AJCC 6th edition) (www.i-kgca.or.kr, 2009 National Gastric Cancer Registration Project). Although various clinical evidence of the efficacy of laparoscopic surgery are presented to these patients, most of such evidences stem from the results of retrospective or cohort studies.[27-30]

Table 3 The number of cases according to T-stage in Korean gastric cancer survey

| | 1995 | 1999 | 2004 | 2009 |
|---|---|---|---|---|
| T1 | 1537 (28.6%) | 2076 (32.8%) | 5196 (47.4%) | 8107 (57.7%) |
| T2 | 1277 (23.8%) | 1659 (26.2%) | 2941 (26.8%) | 3809 (26.8%) |
| T3 | 1733 (32.3%) | 1645 (26.0%) | 2338 (21.3%) | 1799 (12.8%) |
| T4 | 514 (9.6%) | 489 (7.7%) | 491 (4.5%) | 388 (2.8%) |

4) To promote the widespread acceptance of the clinical application of laparoscopy-assisted surgery for locally advanced gastric cancer, it is necessary to establish firm criteria for the conditions best-suited for laparoscopic gastrectomy and D2 lymphadenectomy and to verify its technical safety and oncological effectiveness.

5) The only reliable way to achieve this end is to compare the short-term surgical outcomes (surgery-related parameters such as complication rate, mortality, and operative time) and long-term outcomes (e.g., survival and relapse rates) between laparoscopic and open surgery and to demonstrate the non-inferiority of laparoscopic surgery by conducting a large-scale prospective multicenter randomized clinical study with the participation of institutions that administer laparoscopic gastrectomy as the standard surgical treatment.

6) Such a large-scale phase III study must be preceded by the standardization of surgery as a prerequisite for the quality control of surgery, which is a determinant factor for determining the short- and long-term surgical performance. This standardization of surgery should be established for both laparoscopic and open surgery, and participating surgeons should be selected according to the performance levels as determined by applying standardized evaluation criteria.

## 6. Ethical considerations

1) This study will be carried out upon approval of the Institutional Review Board (IRB) of each study site and in compliance with the Helsinki Declaration and KGCP guidelines.
2) In keeping with the related regulations and guidelines, subjects can participate in the study only after having signed the informed consent form (ICF) in full awareness of the objectives of the study and the risks involved and each subject has the right to withdraw from the study at any time.
3) We will ensure confidentiality of all patient- and study-related information and explain to the subjects the details of compensation.

## 7. Surgical procedures used in the study

1) Arm A: Laparoscopic access to the peritoneal cavity and administration of subtotal gastrectomy with D2 lymph node dissection
2) Arm B: Open access to the peritoneal cavity and administration of subtotal gastrectomy with D2 lymph node dissection

## 8. Study duration

The study is projected to span six years from the IRB approval date: three-year recruitment period and three-year follow-up period (the last enrolled patient will be followed for three years). The study can be extended depending on the enrollment status.

## 9. Selection criteria and sample size calculation

### 9.1 Inclusion criteria

Patients must meet all of the following inclusion criteria to be eligible for the participation in the study:

1) Age between 20 and 80 years.
2) Eastern Cooperative Oncology Group (ECOG) performance status 0 or 1.
3) American Society of Anesthesiologists (ASA) score class I to III.
4) Baseline diagnosis of gastric adenocarcinoma by endoscopic biopsy.
5) Baseline diagnosis of locally advanced gastric cancer without invasion to adjacent organs, although suspicion of invasion beyond the muscularis propria (mp) is acceptable, and without lymph node metastasis or if any, limited to the left gastric artery or the perigastric area.
6) Baseline physical conditions allowing subtotal gastrectomy.
7) Signing of the IRB-approved ICF prior to participation in the study after being sufficiently informed of the objectives and details of this study to make a fully informed and free decision.

### 9.2 Exclusion criteria

Patients who meet any of the following criteria may not be enrolled in the study:

1) Distant metastasis confirmed in the preoperative examination.

2) Past history of gastrectomy.
3) Gastric cancer complications (serious obstruction or perforation).
4) History of chemotherapy or radiotherapy or endoscopic submucosal resection to treat the ongoing gastric cancer.
5) History of surgery, chemotherapy, or radiotherapy for any other primary carcinoma during the past five years (except for cured skin basal cell carcinomas and cervical cancer in situ).
6) Vulnerable conditions (cognitive impairment, ongoing or planned pregnancy).
7) Participation in another ongoing or recent (within the last 6 months) clinical trial(s).
8) Active synchronous double primary cancer.

**9.3 Target patient population and sample size determination**

1) This study aims to verify the non-inferiority of laparoscopic subtotal gastrectomy with D2 lymph node dissection compared with open subtotal gastrectomy in terms of the 3-year RFS rate. Enrolled patients will be randomized into two arms: Arm A (laparoscopic subtotal gastrectomy with D2 lymph node dissection) and Arm B (open subtotal gastrectomy with D2 lymph node dissection). This study will span six years consisting of a three-year recruitment period and a three-year follow-up period (the last enrolled patient would be followed for three years).
2) Drawing on the results of a clinical study reported by *Sakuramoto et al*. in 2007,[31] in which the 3-yr relapse-free survival rate was 72% in patients with locally advanced gastric cancer (Stage II and III) after undergoing subtotal gastrectomy with D2 lymphadenectomy and subsequent adjuvant chemotherapy (hazard rate = 0.11), the hazard rate of Arm B (open subtotal gastrectomy with D2 lymphadenectomy) is assumed to be 0.11 [Cumulative hazard function, $\Lambda(t) = - \log s(t)$, (Hazard rate = 0.11, if t = 3)].
3) Additionally, given that the margin of non-inferiority defined in the above-mentioned study is the hazard ratio (HR) (surgery-only group to adjuvant chemotherapy group) of 1.43, the margin of non-inferiority in this study is assumed to be $HR_0 = 1.43$.
4) In calculating the sample size (PASS 2008),[32] the null hypothesis was formulated as $HR > HR_0$ (HR = hazard rate of Arm A/hazard rate of Arm B) and the alternative hypothesis as $HR < HR_0$. Type I error was set to 0.025 (one-sided), with a target power of 90% in the log-rank test for non-inferiority.
5) Thus, the sample size was determined to be 425 per group, totaling 850 subjects, and the total number of target events (relapses) at 330. Considering a 10% dropout rate, a total of 1050 subjects, for 525 each group, are estimated to be required.

Table 4. Result of sample size by PASS 2008 Numeric Results in Terms of Sample Size

Numeric Results in Terms of Sample Size

| Power | N1 | N2 | N | Equiv Haz Ratio (HR0) | Actual Haz Ratio (HR) | Ref Haz Rate (h1) | Acc-rual Pat'n | Acc-rual Time/ Total Time | Ref Loss | Trt Loss | Ref to Trt | Trt to Ref | Alpha | Beta |
|---|---|---|---|---|---|---|---|---|---|---|---|---|---|---|
| 0.9003 | 425 | 425 | 850 | 1.43 | 1 | 0.11 | Equal | 3/6 | 0 | 0 | 0 | 0 | 0.025 | 0.0997 |
| 0.9001 | 525 | 525 | 1050 | 1.43 | 1 | 0.11 | Equal | 3/6 | 0.1 | 0.1 | 0 | 0 | 0.025 | 0.0999 |

Numeric Results in Terms of Events

| Power | Ref Evts E1 | Trt Evts E2 | Total Evts E | Equiv Haz Ratio (HR0) | Actual Haz Ratio (HR) | Ref Haz Rate (h1) | Acc-rual Pat'n | Acc-rual Time/ Total Time | Ref Loss | Trt Loss | Ref to Trt | Trt to Ref | Alpha | Beta |
|---|---|---|---|---|---|---|---|---|---|---|---|---|---|---|
| 0.9003 | 164.8 | 164.8 | **329.5** | 1.43 | 1 | 0.11 | Equal | 3/6 | 0 | 0 | 0 | 0 | 0.025 | 0.0997 |
| 0.9001 | 164.6 | 164.6 | **329.3** | 1.43 | 1 | 0.11 | Equal | 3/6 | 0.1 | 0.1 | 0 | 0 | 0.025 | 0.0999 |

## 10 Study design

### 10.1 Qualification of participating surgeons and standardization and quality control of surgery

1) Participation in this clinical study is limited to the surgeons meeting the performance standards after participating in the precedence study “Efficacy of Laparoscopic Subtotal Gastrectomy with D2 Lymphadenectomy for Locally Advanced Gastric Cancer (KLASS-02-RCT) and the Standardization of D2 Lymphadenectomy and Surgical Quality Control (KLASS-02-QC)” study.

2) Prior to the selection of the participating surgeons, candidate surgeons will apply for approval to participate in the study to their respective IRB and the IRB-approved surgeons will submit at least three sample surgical videos of open or laparoscopic subtotal gastrectomy.

3) Once the videos submitted are evaluated by a panel comprising domestic and foreign surgeons specializing in gastric cancer, the pre-designated Review Committee will grant participation in the study only to the surgeons meeting the previously established quality standards for D2 lymphadenectomy.

### 10.2 Management of participating sites and patient enrolment

1) The overall management of this study will be carried out by the Regional Clinical Trial Center of Ajou University Hospital, the contract research organization of this study (hereinafter the “CRO”).

2) The CRO will provide the surgeons, whose participation in the study has been approved by the Review Committee, with the application form so that they may obtain approval to participate from their respective IRBs.

3) Upon receiving IRB approval, patient enrollment can commence by obtaining written consent from patients meeting the inclusion criteria. Patient enrollment and management can be achieved using the web-based electronic case report form (eCRF) provided by the CRO.

4) Patient enrollment procedure:
A patient who meets all of the inclusion criteria for randomization and does not meet any of the exclusion criteria will be given detailed explanation about the participation in the study. A signed and dated ICF will be obtained from the patient. The patient will then ben enrolled in the study and randomized to Arm A or Arm B following the eligibility check using the web-based randomization program provided by the CRO (http://clintrial.ajoumc.or.kr/klass02/).

5) Issues related to enrollment:
① In the event that an enrolled patient refuses to participate in the study or the surgeon does not perform the operation within 30 days from the day of enrollment, the patient will be withdrawn from the study.
② In order for a patient to participate in the study after the lapse of 30 days, the selection criteria should be re-applied and a new ICF must be obtained.
③ If an investigator discovers erroneous or duplicate enrollment, the investigator must inform the CRO thereof for correction. If discovered by the CRO, the investigator notified thereof must correct the error and repeat the enrollment procedure (this applies only to patients prior to randomization, i.e., at the screening stage).

6) Closure of patient enrollment:
The patient enrollment will be closed when the number of the enrolled subjects randomized equally to the two arms reaches a total of 1050.

7) Randomization procedure:
① A 1:1 allocation ratio will be applied for randomization with each investigator.
② A baseline number (BN) will be assigned to each patient on signing the ICF. The applicants finally selected as eligible subjects are assigned sequential allocation numbers (ANs) in accordance with the pre-generated randomization table.
③ A randomized block design (Excel 2007) will be implemented for stratified randomization, with each investigator as the stratification variable.
④ The block size will not be disclosed to the investigator to maintain the properties of randomization.
⑤ To enable the use of the eCRF, login details (ID and password) will be set up for each participating surgeon so that he/she can access the assigned account via user authentication (password verification process).

### 10.3 Informed consent process

1) The investigator will provide each recruited subject with sufficient information about the study, in keeping with the Helsinki Declaration and KGCP guidelines, so that he/she can make an informed and voluntary decision about his/her participation in the study.

2) The subject information sheet (SIS) (Annex 1) will be provided to the recruited patients in their respective languages as a detailed information source about the study to help them decide whether or not to provide consent for participation. In addition, they will be given sufficient opportunity to ask questions about the details of the study.

3) The SIS may be read to a subject, but the subject must be given sufficient time to carefully read it and voluntarily decide without any coercion before signing the ICF.

4) Each subject is entitled to withdraw from the study and must be informed that a voluntary withdrawal is possible at any time after the enrollment.

5) The ICF must be approved by the IRB along with this Protocol. The ICF and SIS must be formulated in a manner that is easily understandable by the subjects. ICFs, signed and dated by the subjects, must be stored as an essential study document.

6) Only the IRB-approved ICF is valid. Two original copies of ICF shall be signed and dated: one copy shall be kept by the PI the other copy shall be given to the subject.

7) When drafting the SIS, all necessary elements specified in the KGCP as per Annex 4 of the Pharmaceutical Affairs Act &. Regulation on Safety of Drugs, etc. must be included.

### 10.4 Treatment plan for enrolled patients

Arm A: Laparoscopic subtotal gastrectomy and D2 lymph node dissection
Arm B: Open subtotal gastrectomy with D2 lymph node dissection

1) The following describes the procedure to be administered to each group.

① Surgical procedure for Arm A (laparoscopic surgery)
- Preoperative insertion of a nasogastric tube is optional.
- Prophylactic antibiotics (1st or 2nd generation cephalosporin) are injected within 30 minutes of abdominal incision.
- The type and number of trocars used for the operation are not limited.
- The abdominal cavity is inspected, either laparoscopically or after conversion to open surgery, to determine whether the tumor is resectable.
- Peritoneal washing cytology may be performed with 50 cc saline solution introduced into the pelvic cavity through the trocar.
- The surgeon performs laparoscopic subtotal gastrectomy and D2 lymphadenectomy in accordance with the guideline for the extent of D2 lymphadenectomy during subtotal gastrectomy (Table 1).

- The surgeon may administer additional gastrectomy and reconstruction through an additional small abdominal incision or under laparoscopy, as judged appropriate, after completing the D2 lymphadenectomy.
- The surgeon may perform any reconstruction technique among Billroth-I, Billroth-II, or Roux-en-Y, using any instrument judged appropriate.
- Insertion of a postoperative drainage system is optional according to the judgment by the surgeon.
- Lymph nodes resected are separated from the resected tissues according to the lymph node stations and will be sent to the pathology lab for examination.

Table 5. Guideline of D2 lymph node dissection for locally advanced gastric cancer

| | |
|---|---|
| 1. Total omentectomy | 4d |
| 2. Division of left gastroepiploic artery | 4Sb |
| 3. Appropriate extent of No.6 lymph node (LN) dissection | 6 |
| 4. Appropriate extent of No.5 LN dissection | 5 |
| 5. Appropriate extent of No.12a LN dissection | 12a |
| 6. Appropriate extent of No.8a LN dissection | 8a |
| 7. Appropriate extent of No.9 LN dissection (resection of the celiac plexus is not necessary) | 9 |
| 8. Appropriate extent of No.7 LN dissection | 7 |
| 9. Appropriate extent of No.11p LN dissection | 11p |
| 10. Prevention of pancreatic injury during suprapancreatic LN dissection | |
| 11. Appropriate extent of No.1 and 3 LN dissect ion | 1, 3 |

② Surgical procedure for Arm B (open surgery)

- Preoperative insertion of the nasogastric tube is optional.
- Prophylactic antibiotics (1st or 2nd generation cephalosporin) are injected within 30 minutes of abdominal incision.
- Incision is made from the xiphoid process to just above the umbilicus.
- The abdominal cavity is inspected to determine whether the tumor is resectable.
- Peritoneal washing cytology may be performed with 50 cc saline solution introduced into the pelvic cavity after the incision.
- The surgeon performs subtotal gastrectomy and D2 lymphadenectomy following the guidelines in the same manner as in laparoscopic surgery.
- Reconstruction and insertion of a postoperative drainage system are performed in the same manner as in laparoscopic surgery.
- Lymph nodes are separated from the resected tissues according to the lymph node stations and sent to the pathology lab for examination.

2) Clinical study methods based on intraoperative findings

① If the investigator judges that gastrectomy/lymphadenectomy is impossible or inappropriate, based on the intraoperative findings (applicable to both laparoscopic and open procedures), the operation is discontinued and the patient will be withdrawn from this study.

② Other clinical study methods to be applied depending on intraoperative findings are described under Section 12: Criteria for the continuation of the study.

### 10.5 Postoperative patient management

1) Patient management will be achieved in accordance with the common clinical practice guidelines as described in the Protocol.

2) To prevent postoperative thrombosis, the investigator may use a mechanical leg compression device, elastic stockings, or anticoagulants before and immediately after surgery, according to necessity.

3) The prophylactic antibiotics administered before surgery may be used postoperatively for an appropriate period of time.

4) Postoperative resumption of oral intake of food after a certain period of fasting may occur in the order of water, liquid meals, and soft diet, as judged appropriate by the investigator.

5) Postoperative pain control is achieved by appropriate patient-controlled analgesia (PCA) pumps and additional medication can be administered if requested by the patient.

### 10.6 Postoperative care depending on pathological outcomes

1) Adjuvant chemotherapy based on 5-FU will be administered within 4 to 6 weeks after surgery to patients diagnosed with Stage II or Stage III disease as the postoperative pathological outcomes and found to meet the following criteria in basic blood chemistry tests:

① Intraoperative and pathological findings of radical resection
② No evidence of early relapse on computed tomography (CT) images obtained before chemotherapy
③ ECOG performance status of 0 or 1
④ WBC $\geq 3000/mm^3$ with neutrophils $>1500/mm^3$
⑤ Platelet count $\geq 100{,}000/mm^3$
⑥ Hb ≥9 g/dL
⑦ Total bilirubin ≤1.5 × upper reference range
⑧ AST ≤2.5 × upper reference range
⑨ Serum creatinine ≤1.5 × upper reference range

2) In accordance with the results of a prospective multicenter study reported by Sakuramoto et al. in 2008,[31] one-year TS-1 monotherapy is considered to be the standard adjuvant chemotherapy. Anticancer agents based on 5-FU can be administered concomitantly, whereby the dose and duration are determined by the method of administration of the anticancer drugs. Adjuvant chemotherapy may be administered at the investigator’s discretion taking into account the patient’s conditions and subject to his/her consent. The mode of administration may be adjusted at any time, including dose reduction, discontinuation, and extension of duration.

### 10.7 Efficacy and safety assessment

1) Efficacy assessment parameters
   To evaluate the efficacy of laparoscopic submucosal gastrectomy and D2 lymphadenectomy for locally advanced gastric cancer, the following parameters will be measured.
   ① 3-year RFS and OS of the patients subjected to laparoscopic and open submucosal gastrectomy and D2 lymphadenectomy
   ② Histopathological findings after laparoscopic and open gastrectomy and D2 lymphadenectomy
   ③ Early and late complications, within and after postoperative day 21, respectively, of the patients subjected to laparoscopic and open submucosal gastrectomy and D2 lymphadenectomy
   ④ Postoperative recovery profile (bowel movement, oral intake of food, length of hospital stay, etc.), and quality of life (EORTC C-30 and Sto-22 scores at baseline, postoperative day 25, and year 1) of the patients subjected to laparoscopic and open submucosal gastrectomy and D2 lymphadenectomy

2) Safety assessment parameters
   To evaluate the safety of laparoscopic submucosal gastrectomy and D2 lymphadenectomy for locally advanced gastric cancer, the following parameters will be measured.
   ① Early postoperative complications with onset within three weeks after surgery
   ② Late postoperative complications with onset three weeks after surgery
   ③ Assessment of postoperative adverse events other than anticipated complications
   ④ Postoperative mortality (90-day all-cause mortality after surgery)

## 11. Assessment items, clinical exam items, and observational assessment method

### 11.1 Baseline assessment items

The time period from the signing of the ICF to surgery, during which the preoperative study procedures are carried out, including the completion of the subject eligibility assessment, is referred to here as the baseline. The investigator screens and selects individual patients based on the application of the inclusion and exclusion criteria.

1) Diagnosis of gastric adenocarcinoma: The patient should be assessed for the eligibility of gastrectomy before surgery and diagnosed with gastric adenocarcinoma through biopsy.

2) Basic patient Information collection: The patients enrolled into study are interviewed by interviewers before surgery.
   ① Age, sex, height, weight, medical history, medication history
   ② ECOG performance status, ASA score, comorbidities, drinking history, smoking history
   ③ History of abdominal surgery, other primary cancer, and endoscopic resection for other gastric lesions

3) The following criteria shall be applied when rating the ECOG performance status:

① 0: Fully active, able to carry on all daily activities without any restriction
② 1: Able to carry out light activities, active for more than 50% of waking hours without any restriction
③ 2: Capable of self-care, active for less than 50% of waking hours
④ 3: Capable of only limited self-care, mostly confined to a wheelchair
⑤ 4: Completely incapable of self-care, mostly confined to bed
⑥ 5: Dead

4) The following criteria shall be applied when determining the ASA score:
① Class 1: A healthy patient with no systemic disease
② Class 2: A patient with mild/moderate systemic disease due to surgery or comorbid conditions
③ Class 3: A patient with severe systemic disease that restricts daily life
④ Class 4: A patient with severe systemic disease that is a constant threat to life
⑤ Class 5: A moribund patient with the 24-h mortality rate of 50% regardless of operation
⑥ Class 6: A declared brain-dead patient whose organs are being removed for donor purposes
⑦ Emergency surgery (E): The addition of "E" to the ASA physical status classification means that the patient requires emergency surgery.

5) The following criteria shall be applied when assessing comorbidities:

Table 6. Charlson index component (modified)

| Comorbid condition |
|---|
| Myocardiac Infarct or ischemic heart disease |
| Congestive Heart Failure |
| Hypertension |
| Peripheral Vascular Disease |
| Cerebrovascular disease |
| Dementia |
| Chronic pulmonary disease |
| Connective tissue disease |
| Ulcer disease |
| Mild liver disease (hepatitis or elevated liver enzymes) |
| Diabetes without end organ damage |
| Hemiplegia |
| Moderate or severe renal disease |
| Diabetes with end organ damage |
| Leukemia |
| Lymphoma |
| Moderate or severe liver disease (liver cirrhosis) |
| Metastatic solid tumor |
| Any other malignant tumor |
| AIDS |

– Comorbid disorders are examined in two categories of previous and current treatments.
– Also examined are concomitant medications for the treatment of co-morbidities.

6) Diagnostic staging tests
   ① Blood test (complete blood cell count, blood chemistry, coagulation test):
   ** WBC, Hb, Hct, Platelet, Neutrophil, BUN, Cr, ALT, AST, amylase, Total bilirubin, PT, aPTT, INR, hCG (urine or serum)
   ② Chest radiography
   ③ Abdominopelvic contrast-enhanced CT: Other tests may be added according to each patient's needs, such as Positron emission tomography-CT (PET-CT), abdominal Magnetic resonance imaging (MRI), endoscopic ultrasound biopsy, image-guided biopsy, etc.
   ④ Tumor markers (CEA, CA19-9): Test results may be used as reference data for determining postoperative relapse.
   ⑤ Evaluation of EORTC-STO22 and C30:
   – The questionnaire surveys will be conducted after receiving EORTC certification to use the results as reference data to compare the preoperative and postoperative quality of life.
   – The questionnaire surveys will be conducted by an independent third party within one week before surgery to ensure reliable assessments.

**11.2 Assessment items on the operation day**

On the day of the operation, in which the subject undergoes the scheduled gastric cancer surgery using the operative technique for Arm A or Arm B to which each subject was preoperatively randomized. The following assessment items will be observed peri- and intraoperatively and documented in the operative report and data forms:

1) Anesthesia time (time from start and end of anesthesia care).
2) Operation time (from abdominal incision to skin suture).
3) Incision length.
4) Intraoperative evaluation of tumor cell invasiveness and metastasis.
5) Resection technique used:
   If gastrectomy is not performed, the reason should be documented. If radical gastrectomy is performed instead of distal subtotal gastrectomy, the reason should be documented.
6) Extent of lymph node dissection:
   The extent of lymph node dissection and identification of lymph node stations should be documented. If D2 lymphadenectomy is not performed, the reason should be documented.
7) Reconstruction:
   The reconstruction technique used (Billroth-I, Billroth-II, or Roux-en Y) and the reason for the choice of should be documented. If a different reconstruction technique has been used, the type and reason should be documented. In the case of laparoscopic surgery, it should also be documented whether intraperitoneal anastomosis or small incision was performed.
8) Gastrectomy with combined resection of other organs:
   In the event of combined resection of other organs, the reason should be documented.
9) Macroscopic curative resection

If no macroscopic curative resection could be performed, the reason should be documented.

10) Intraoperative blood loss and transfusion
① Blood loss: blood absorbed is measured in cc, taking account of the weight of gauze.
② Transfusion: the amount of red cells and whole blood transfused is measured in cc.

### 11.3 Assessment items during the postoperative hospital stay

In the period from postoperative day 1 to discharge, the recovery profile and complications are assessed.

1) After surgery, the investigator will assess the patient's recovery (passage of gas) once a day.

2) Recovery parameters:
– Daily check and documentation: oral feeding, bowel movement, pain score (on a 10-point scale).
– Check and document on postoperative day 1 (+ 1 day) and day 5 (± 1 day): blood test**.
** WBC, Hb, Hct, Plt, Neutrophil, BUN, Cr, ALT, AST, amylase, total bilirubin

3) Determination of complications: The observational criteria for early postoperative complications presented by the KLASS study group will be applied for daily check for complications along with type and severity, and appropriate care will be sought and administered.[11]

Table 7. Evaluation of postoperative morbidity

| Morbidity | Onset POD ( ) | Management (Please enter) | Grade |
|---|---|---|---|
| 0: No complication | | | |
| 1: Wound infection | | | |
| 2: Fluid collection/abscess | | | |
| 3: Intra-abdominal bleeding | | | |
| 4: Intraluminal bleeding | | | |
| 5: Postoperative ileus | | | |
| 6: Anastomosis stenosis | | | |
| 7: Leakage | | | |
| 8: Pancreatitis or fistula | | | |
| 9: Pulmonary | | | |
| 10: Urinary | | | |
| 11: Renal | | | |
| 12: Hepatic | | | |
| 13: Cardiac | | | |

| | | | |
|---|---|---|---|
| 14: Endocrine | | | |
| 15: Others ( ) | | | |

(POD: postoperative day)

4) Assessment of the discharge status and the actual discharge date:
If the patient’s condition satisfies the discharge criteria, it should be documented, and if it does not coincide with the actual discharge date, the reason should be investigated.

### 11.4 Assessment items on postoperative day 25

As for the patients who could not be discharged or have been re-admitted by the time point POD 25, the reasons for the prolonged hospital stay and readmission will be documented and the following items will be investigated. Patients discharged will visit the study site for follow-up on POD 25 ± 4 for the same assessment.

1) Continued hospital stay until POD 21: if affirmative, the reason for the prolonged stay.
2) Re-admission: if affirmative, the reason for re-admission.
3) Final check for early postoperative complications.
4) Measurement of POD 25 quality of life score.
5) Check for postoperative pathological findings.
– Postoperative pathological outcomes
① The extent of primary tumor invasion, lymph node metastasis, and other histological characteristics.
② Distance from the resection margin to the tumor.
③ Rate of positive peritoneal washing cytology.
④ Other biopsy results.
6) If a patient requires adjuvant chemotherapy according to pathological outcomes, administration of anticancer drugs will be determined after testing the suitability of the given drugs for their personalized use.

### 11.5 Assessment items at postoperative months 3, 6, 9, 12, 15, 18, 21, 24, 27, 30, 33, and 36

According to the study calendar (study flow sheet), patients will be followed up for relapse and survival at postoperative months (POM) 3, 6, 9, 12, 15, 18, 21, 24, 27, 30, 33, and 36. However, if the visiting interval is too taxing for the patient, the investigator can adjust the interval at his/her discretion. However, follow-up visits at POM 6, 12, 18, 24, 30, and 36 are mandatory.

For the quarterly follow-up visits, a time slot of ±14 days is allowed.

- For visits at POM 3, 6, 9, 12, 15, 18, 21, 24, 27, 30, and 33, a visit on the scheduled date ±30 days is not regarded as noncompliance of visiting date.
- For the last visit at POM 36, a 12-month grace period is provided; a case conclusion documented by POM 48 is not therefore regarded as noncompliance of visiting date.

1) Patients in the RFS analysis group will be subjected to the following tests to be checked for relapse during the study period until the relapse is confirmed.
   ① Performance status and physical examination
   ② Weight
   ③ Tumor markers: CEA, CA 19-9
   ④ Chest X-ray and contrast-enhanced CT of the upper abdomen and pelvis (CT without contrast agent in patients showing adverse reactions to contrast agents): first administration at POM 6 and every six months thereafter
   ⑤ During the visit at POM 3, 9, 15, 21, 27, and 33, neither contrast-enhanced CT of the upper abdomen and pelvis nor abdominal ultrasound is administered, or either analysis is administered optionally.
   ⑥ Upper gastrointestinal endoscopy: first administration at POM 12 and every 12 months thereafter
   ⑦ Blood test **: (complete blood cell count, blood chemistry)

   ** WBC, Hb, Hct, Plt, Neutrophil, BUN, Cr, ALT, AST, Total bilirubin
   *** Blood test items at the end of study/premature termination
   WBC, Hb, Hct, Plt, Neutrophil, BUN, Cr, ALT, AST, Total bilirubin

   PET, CT, MRI, diagnostic laparoscopy, and radiologic biopsy may be performed to confirm relapse according to the results of WBC, Hb, Hct, Plt, Neutrophil, BUN, Cr, ALT, AST values, should be recorded.

2) Interview to check for the occurrence of late complications during follow-up examination (If affirmative, the case will investigated according to section 11.6 Assessment items related to late complications).

3) Check for relapse/relapse-free survival (in the event of relapse, the case will be investigated according to section 11.7 Relapse-related assessment items).

4) Check for survival/death (in the event of death, the case will be investigated according to section 11.8 Death-related assessment items).

5) Investigation of issues related to adjuvant chemotherapy (in the event of using adjuvant anticancer drugs, the case will be investigated according to section 11.9 Assessment items related to adjuvant chemotherapy).

6) Quality of life questionnaire survey
   The EORTC-STO22 and C30 quality of life questionnaire surveys administered at baseline will be re-administered at POM 12.

**11.6 Assessment items related to late complications**

1) Surgical complications with late onset (>21 days postoperatively) will be observed.

2) Surgical complications occurring between two follow-up appointments will be confirmed and documented at the subsequent appointment.

3) Late complications will be investigated according to the scheme presented in Table 8 on confirming items related in-patient care (including data derived from other institutions).

Table. Late complication during follow up period

| Late complications | 0:no, 1-8:yes | Management |
|---|---|---|
| 1. Int. obstruction | POD ( )months | 1: Conservative treatment<br>2: Endoscopy or intervention<br>3: Reoperation<br>4: 기타 ( ) |
| 2. Stenosis | POD ( )months | 1: Conservative treatment<br>2: Endoscopy or intervention<br>3: Reoperation<br>4: 기타 ( ) |
| 3. Chronic wound complica-tion | POD ( )months | 1: Conservative treatment<br>2: Endoscopy or intervention<br>3: Reoperation |
| 4. Others( ) | POD ( )months | 1: Conservative treatment<br>2: Endoscopy or intervention<br>3: Reoperation<br>4: 기타 ( ) |

**11.7. Relapse-related assessment items**

1) In the event of relapse detection, timing and pattern of relapse will be documented as indicated below and the patient will be followed for survival.

2) Date of relapse detection during a follow-up examination.

3) Site of relapse: The location of relapse detection is categorized into remnant stomach (including anastomosis), peritoneum, liver, lung, bone, ovary, regional lymph node, distal lymph node (including aorta and retroperitoneum), and others (to be specified). If two or more places are affected, all must be indicated.

4) Diagnostic confirmation of relapse: examination modalities used for relapse detection must be documented from the physical examination, CT, ultrasound, endoscopy, PET-CT, MRI, bone scan, If two modalities are combined, both must be indicated.

5) Relapse treatment plan: On confirming recurrent gastric cancer, initial treatment will be administered in the categories of chemotherapy, radical resection, radiotherapy, and other treatment. If two or more treatment options are combined, all must be indicated. In the case of

chemotherapy, type of anticancer drugs administered must be specified and in the case of radiotherapy, the dose should be estimated and indicated.

### 11.8 Survival- and death-related assessment items

1) Mortality will be monitored and evaluated in all patients except for those that have withdrawn from the study.
2) If a patient's survival or death cannot be confirmed through visit or telephone contact, the related information can be retrieved via the National Cancer Registration Program managed by the National Cancer Center. The consent to the information retrieval must be obtained from the subjects in advance through the relevant SIS-ICF.
3) Until the date of the last visit of the last subject, all subjects' survival status may be monitored through visit or phone call every six months, even after the completion of the study at POM 36. If death is confirmed, the following items will be confirmed or assessed:
   ① Date of death
   ② Association with early postoperative complications
   ③ Cause of death

### 11.9 Assessment items related to adjuvant chemotherapy

1) Type of adjuvant anticancer drugs.
2) Date of first administration of adjuvant anticancer drugs.
3) Reason for post-planning dose reduction or non-administration of anticancer drugs, if applicable.
4) Duration of administration of the planned adjuvant anticancer drugs.

### 11.10 Study flow sheet (study calendar)

| Events/Assessment Items | Screening/ Baseline[1] | Surgery | POD 1 – Discharge | POD 25 | POM 3 –36 | Study end/premature termination | Follow-up after the study end |
|---|---|---|---|---|---|---|---|
| Visit | | 0 | | | POM 3,6,9,12,15,18,21,24,27,30,33,36 | | Every 6 months after the study end |
| Visiting time slot | | | | ±4 d | ±14 d | | |
| Consent procedure[2] | V | | | | | | |
| Exclusion criteria check | V | | | | | | |
| Randomization[3] | V | | | | | | |
| Patient demographics | V | | | | | | |
| Medical history, ASA | V | | | | | | |
| ECOG | V | | | V | V | V | |
| Physical exam/weight | V | | | V | V | V | |

| Events/Assessment Items | Screening/ Baseline[1] | Surgery | POD 1 – Discharge | POD 25 | POM 3 –36 | Study end/premature termination | Follow-up after the study end |
|---|---|---|---|---|---|---|---|
| Visit | | 0 | | | POM 3,6,9,12,15,18,21,24,27,30,33,36 | | Every 6 months after the study end |
| Visiting time slot | | | | ±4 d | ±14 d | | |
| Past medical/medication history | V | | | | | | |
| Complete blood test | V | | V[4] | | V | V | |
| Blood chemistry test | V | | V[5] | | V | V | |
| Blood coagulation test | V | | | | | | |
| Tumor marker test[6] | V | | | | V | V | |
| Chest X-ray, abdominopelvic CT | V | | | | V[7] | V | |
| (or) abdominal ultrasound | | | | | | | |
| Upper gastrointestinal endoscopy | | | | | V[8] | | |
| Surgery | | V[9] | | | | | |
| Recovery assessment[10] | | | V | | | | |
| Check for early complications | | | V | V | | | |
| Check for late complications | | | | | V | V | |
| Pathological outcomes[11] | | | | V | | | |
| Chemotherapy (if applicable)[12] | | | | V | V | | |
| Adverse events[13] | | V | V | V | V | | |
| Quality of life measurement[14] | V | | | V | V[15] | | |
| Relapse/Survival status | | | | | V | V | V[16] |
| Case conclusion[17] | | | | | | V | |

(POD: postoperative day; POM: postoperative month)

1 A period of up to three months is allowed between screening testing and surgery.
2 The ICF must be re-obtained if surgery does not take place after 30 days from signing the ICF.
3 Randomization is conducted after the completion of all screening tests.
4 Complete blood test is performed on POD 1 (± 1 day) and POD 5 (± 1 d).
5 Blood chemistry test is performed on POD 1 (± 1 day) and POD 5 (± 1 d).
6 CEA and CA19-9.
7 Chest X-ray and abdominopelvic CT are performed at POM 6 and every 6 months thereafter (POM 6, 12, 18, 24, 30, and 36). At POM 3, 9, 15, 21, 27, and 33, neither CT nor abdominal ultrasound is administered, or either one is administered optionally.

[8] Upper gastrointestinal endoscopy is performed at POM 12 follow-up visit and every 12 months thereafter. (POM 24 and 36).
[9] Surgery for Arm A or Arm B is performed as randomized.
[10] Oral intake of food, passage of flatus (intestinal gas), degree of pain, and discharge are checked.
[11] The final pathological outcomes are checked to evaluate to the course of the study.
[12] In cases where chemotherapy is administered, the effects of anticancer drugs and adverse events are checked.
[13] Adverse events are assessed up to POM 12.
[14] EORTC-ST022 and C30 evaluation sheets are drafted.
[15] Quality of life is assessed only during the POM 12 visit.
[16] Subjects who completed the POM 36 visit are followed up every six months.
[17] Case conclusion is drawn by choosing one of the following: Yes, No, and Expected last visit.

## 12. Criteria for the continuation of the study

### 12.1 Withdrawal criteria

1) Refusal to participate in the study after signing of the ICF and randomization.
2) Surgeon’s judgment based on intraperitoneal examination that gastrectomy is impossible due to invasion into adjacent organs or distant metastasis.
3) No surgery 30 days after the signing of the ICF.

### 12.2 Conversion from laparoscopic to open surgery: principles and procedures

1) Conversion to open surgery is defined as a change from laparoscopic to an open procedure without terminating lymphadenectomy and completing the operation by additionally performing steps of open surgery.
2) Conversion to open surgery will take place when the operating surgeon judges it necessary in inevitable circumstances such as need for combined resection due to invasion into adjacent organs, peritoneal adhesions, and excessive bleeding.
3) Conversion to open surgery will not result in withdrawal from the study. The related cases will be included in the intention-to-treat (ITT) analysis set or in the full analysis set (FAS) (see section 15.1 for the definition of the ITT analysis set or FAS) for analysis purposes.

### 12.3 Conversion from distal subtotal gastrectomy to total gastrectomy

1) Conversion from laparoscopic or open distal subtotal gastrectomy to total gastrectomy will take place when judged by the operating surgeon that distal subtotal gastrectomy is not sufficient to secure proximal resection margin, i.e., negative gastric cancer cells, contrary to the baseline scenario.
2) Conversion to total gastrectomy will not result in withdrawal from the study. The related cases will be included in the ITT analysis set or the FAS (see section 15.1 for the definition of the ITT analysis set or FAS) for analysis purposes.

### 12.4 Cases requiring combined resection of adjacent organs for radical resection

1) Combined resection of adjacent organs can be performed when judged by the operating surgeon that gastrectomy alone is not sufficient to secure the proximal resection margin, i.e., negative gastric cancer cells, contrary to the baseline scenario.

2) Combined resection of adjacent organs will not result in withdrawal from the study. The related cases will be included in the ITT analysis set or the FAS (see section 15.1 for the definition of the ITT analysis set or FAS) for analysis purposes.

## 13. Termination of the study

1) The study may be terminated by the investigator when an unacceptable problem arises.

2) Consent withdrawal by the subject

## 14. Subject safety protection and appropriate responses to adverse events

1) The PI and Sub-I have the obligation to accurately analyze and thoroughly understand the Protocol so that they can take adequate measures to manage unanticipated adverse events and write reports accordingly as required, take preparatory measures such as training of the study team, and provide insurance coverage of unanticipated adverse events (LIG Life Insurance Co., Ltd.). In the event of injury directly related to the study procedure as per the Protocol, the subject affected will be compensated within the scope of the insurance coverage provided by the indemnity insurance in addition to appropriate medical care.

2) The study will be conducted in compliance with the KGCP guidelines.

3) The investigator will take appropriate measures to manage the adverse events encountered during the course of the study and will report cases of unexpected serious adverse events to all participating sites (see Section 18. Adverse event reporting).

4) Tests and treatments for adverse events will be carried out in accordance with customary medical practices.

5) In the event of emergency, each study site will take measures in accordance with its routine practices for emergencies.

## 15. Analysis set and statistical analysis plan

### 15.1 Analysis sets

1) Efficacy analysis set

(1) ITT analysis set
All enrolled patients, except for those meeting the following withdrawal criteria, will be included in the ITT analysis to be performed to compare the secondary efficacy endpoints of the study, namely recovery, quality of life, and 3-year OS.

1. Randomized, but not operated patients (patients operated with the other surgical procedure after refusing to undergo the allocated procedure are included in the analysis).
2. Operated patients, on whom no resection could be performed.

(2) Full analysis set (FAS)

All randomized patients, except for those listed in the following, will belong to Arm A (laparoscopic subtotal gastrectomy and D2 lymphadenectomy) and Arm B (open subtotal gastrectomy and D2 lymphadenectomy) as the FAS for the comparison of the primary efficacy endpoint, namely 3-year RFS, and the secondary efficacy endpoints, namely recovery, quality of life, and 3-year OS.

1. Randomized, but not operated patients (patients operated with the other surgical procedure after refusing to undergo the allocated procedure are included in the analysis).
2. Operated patients in which no resection could be performed.
3. Operated patients in which resection could be performed, but macroscopic and histological findings could not confirm radical resection or combined resection of additional peritoneal lesions (synchronous peritoneal carcinomatosis from gastric cancer) that were occult on baseline exams.
4. Operated patients with distant metastases, which were not detected intraoperatively but were confirmed in POD 21 pre-chemotherapy exam or physical exam due to other symptoms

– Apart from these circumstances, Protocol non-compliance including conversion from laparoscopic to open surgery, adherence to the follow-up schedule, and premature termination of the study have no influence on the FAS.

(3) Per-protocol (PP) analysis set

The PP analysis set is a subset of the FAS defined by excluding the following subjects:

1. Patients who received, at the investigator's discretion or patient characteristics, the surgical procedure of the Arm to which they had not been allocated (including the conversion from laparoscopic to open surgery).
2. Patients who received combined resection of adjacent organs or total (instead of subtotal) gastrectomy based on intraoperative findings.
3. Patients lost to follow-up for reasons other than death.

– Analysis of the PP analysis set will be performed for auxiliary purposes if the difference in the number of patients between FAS and PP analysis set is 10% or more.

2) Safety analysis set

The SAS is composed of all FAS patients subdivided according to the surgical procedures they received. Safety analysis includes postoperative complications and mortality.

**15.2 Statistical methods**

1) Statistical analysis of primary endpoint of efficacy

The primary objective of this study is to establish the non-inferiority of laparoscopic subtotal gastrectomy with D2 lymph node dissection compared with open subtotal gastrectomy with D2 lymph node dissection. The primary endpoint of efficacy is the 3-year RFS. The HR for RFS comparing laparoscopic versus open surgery will be used to test the hypothesis that laparoscopic surgery is not inferior to conventional open surgery (margin of non-inferiority $HR_0 = 1.43$).

2) Statistical analysis of secondary endpoint of efficacy

① The Kaplan-Meier curve analysis and the log rank test will be performed to compare the 3-year RFS between Arm A (laparoscopic subtotal gastrectomy with D2 lymph node dissection) and Arm B (open subtotal gastrectomy with D2 lymph node dissection).

② Descriptive statistics will be used for the analysis of the postoperative recovery profile. Student t-test will be performed to compare the number of days to first postoperative flatus and the length of hospital stay between the laparoscopic surgery group (Arm A) and the open surgery group (Arm B).

③ The postoperative change in the degree of pain will be compared between the two surgery groups every morning using the repeated measured ANOVA test.

④ Differences in the final pathological outcomes between the two groups will be verified by comparing each of the following characteristics.

1. Invasiveness of the primary tumor, lymph node metastasis, and other histological characteristics
2. Distance from the resection margin to the tumor
3. Rate of positive peritoneal washing cytology
4. Other biopsy results

⑤ To compare the quality of life of both groups, EORTC QLQ-C30 and EORTC QLQ-STO22 will be analyzed at baseline, POD 21, and POM 12 using the following methods:

1. Analysis of EORTC QLQ-C30 will be performed in the following modules: five functional scales (physical, role, emotional, cognitive, and social functioning), three symptom scales (fatigue, pain and nausea, and vomiting), a global health status, and six single items.
2. Analysis of EORTC QLQ-STO22 will be performed in the following modules: five gastric surgery-related scales (dysphasia, eating restriction, pain, reflux, and anxiety) and four single items (dry mouth, body image, taste, and hair loss).
3. Comparison between the laparoscopic and open surgery groups will be made using a multilevel mixed-effects linear regression model.

3) Statistical analysis of safety endpoint

① The difference in the frequency of developing surgical complications between the laparoscopic and open subtotal gastrectomy groups will be analyzed using the Chi-square test.

② The 90-day all-cause mortality of the patients included in the safety analysis set will be monitored and compared between the two surgery groups and analyzed using the Chi-square test.

③ The frequency of deaths and complications from non-surgical causes will be monitored and the intergroup differences will be analyzed using the Chi-square test

4) Handling of missing data

Missing data in the efficacy and safety endpoint analysis will not be replaced.

### 15.3 Analysis time points and the final analysis

1) Timing of analysis
No intergroup comparison of parameters assessing the efficacy of their respective surgical approaches, such as 3-year RFS, OS, and recovery profile, will be made during the course of the study except when approved by the Steering Committee.

2) Interim analysis
① The purpose of this study is to establish the non-inferiority of laparoscopic subtotal gastrectomy with D2 lymph node dissection compared with open subtotal gastrectomy with D2 lymph node dissection in terms of 3-year RFS.
② An interim analysis of the primary endpoint of efficacy, namely the 3-year RFS rate, will be performed, using the Haybittle-Peto interim monitoring boundary, when the number of relapses reaches 165, which is 50% of the total relapses (n = 330) calculated from the number of target subjects. Drawing on Freidlin et al. (2009),[33] the first interim analysis will be based on 0.001, and the final analysis on 0.025.

3) Final analysis
① Final analysis will be performed by the CRO on completion of the 3-year follow-up of all enrolled patients by analyzing the entire data set collected. The CRO will draft the final analysis report and summary.
② The report and summary will be submitted to the study participants and the Steering Committee of the Korean Laparoscopy Gastrointestinal Surgery Study Group (KLASS).
③ The steering committee will draft the Study Report based on the contents of the final analysis report submitted by the CRO and end the study.
④ After the end of study, all basic data and essential documents will be transferred to the Coordinating PI. The Coordination PI will provide data, after consultation with the Steering Committee, when requested by relevant research groups and institutions.

## 16. Efficacy assessment criteria and assessment methodology

### 16.1 Efficacy determination

- Relapse over 36 postoperative months is the outcome measure for the evaluation of the primary objective of this study, i.e., the 3-year RFS rate, is relapse.
- The secondary efficacy endpoints, namely the 3-year OS, postoperative recovery, and quality of life, may serve as the auxiliary criteria for efficacy assessment.

### 16.2 Evaluation of the efficacy endpoints

1) Relapse-free survival
① "Event" of RFS is defined as the confirmed relapse in the FAS after POD 21. "Censored" is defined as a patient lost to follow-up for any reason or a patient who has not experienced relapse over the follow-up period.
② Any of the following is considered an "event" (relapse):
1. Definite diagnosis of asymptomatic relapse in the follow-up exam:

1) Radiologist's reading of endoscopic biopsy.
2) Radiologist's reading of chest or neck CT scan additionally performed when relapse is suspected in regions other than the abdominopelvic regions.
3) Radiologist's reading of PET CT or liver ultrasound and MRI additionally performed when abdominopelvic CT, abdominal ultrasound, or neck and chest CT fail to provide definite diagnosis.
4) Colonoscopy, gastroscopy, or laparoscopy-directed biopsy when peritoneal relapse is suspected, but imaging findings fail to provide definite diagnosis.
5) Confirmatory biopsy for recurrent legions detected by surgical biopsy or image-assisted biopsy.
6) Tumor markers such as CEA and CA19-9 may be used for additional exam or definite diagnosis.

2. Procedure for a relapse suspected, but not confirmed by the above exams:
   1) Administration of the follow-up exam ahead of schedule at the discretion of the PI.
   2) Radiologist's judgment on lesions appearing enlarged in comparison to the previous follow-up exam.
   3) Tumor markers such as CEA and CA19-9 may be used for additional exam or definite diagnosis.

3. Procedure for a clinically suspected relapse:
   1) The relevant follow-up exam can be administered regardless of schedule in case of recurrent symptoms confirmed in physical examination, such as a palpable mass, weight loss, intestinal obstruction, or pain at a specific site.
   2) Diagnosis of the first suspected relapse can be made according to the diagnostic principles described under point 1 above, and in case of an unconfirmed relapse event, the relevant follow-up exam can be administered regardless of schedule according to the diagnostic principles described under point 2 above.

4. Relapse reporting
   1) The investigator must enter a confirmed relapse into the eCRF immediately after a definitive diagnosis along with the following details:
   2) Date of confirmed relapse event:
      ① Date of radiological imaging or histopathological exam when the recurrence is diagnosed on follow-up exam.
      ② Date of the first radiological imaging on the follow-up exam administered ahead of schedule on suspicion of relapse for a definitive diagnosis.
   3) Place of relapse: remnant stomach (including anastomosis), peritoneum, liver, lung, bone, ovary, regional lymph node, distal lymph node (including aorta and retroperitoneum), and others (multiple selections are available).

4) Confirmatory exam of relapse: abdominopelvic CT, thoracic and cervical CT, liver MRI, PET-CT, laparoscopy, upper gastrointestinal endoscopy, lower gastrointestinal endoscopy, others (multiple selection is available).

5) Relapse management plan: chemotherapy, radiotherapy, curative/radical surgery, conventional surgery (multiple selection is available).

2) Overall survival

① "Event" of OS is defined death of any of the enrolled patients (except for those withdrawn from the study) confirmed by study site records, phone, or information retrieved from the relevant national database. "Censored" is defined as a patient alive or lost to follow-up for any reason over the follow-up period.

② Death can be documented immediately if it occurs at the study site where the patient has been followed.

③ The OS assessment period coincides with the study period.

3) Postoperative recovery profile

① Time to postoperative recovery of bowel function:
The day of first postoperative flatus (passage of intestinal gas) will be monitored every morning and recorded.

③ Postoperative change in pain level:
- Daily check of self-reported pain level (questionnaire rated on a 10-point scale)
- The postoperative pain control device, type of additionally administered analgesics, and frequency of use will be monitored and recorded.

③ Postoperative hospital stay
Hospital discharge will be recommended to the patient if the following conditions are met, and the date of discharge recommendation will be recorded.

1. Stable vital signs.
2. Self-reported pain level requiring no additional intravenous infusion of analgesics.
3. No noticeable gastrointestinal symptoms such as abdominal pain, nausea, and vomiting after three consecutive oral intake of soft food.

Patient's refusal of hospital discharge for any reason, even though all the above criteria are met, will be recorded and the actual discharge date will be verified and recorded.

4) Postoperative quality of life
The patient's quality of life will be assessed at baseline, on POD 25, and at POM 12, using the scales provided in the EORTC questionnaires.

## 17. Criteria for safety assessment and methods of assessment and reporting

Of the complications related to the surgical procedures performed in this study, serious complications and deaths associated with lymphadenectomy will be reported.

### 17.1 Methods of postoperative complication assessment and reporting

The postoperative adverse events occurring in this study will be assessed in two categories of early postoperative complications (≤POD 21), along with subsequent prolonged hospital stay and re-admission, and late postoperative complications (>POD 21).

- Complications occurring during hospital stay will be documented (type and POD) and managed immediately.
- Complications occurring after hospital discharge will be documented (type and POM) and managed during admission or on out-patient follow-up exam.

1) Early postoperative complications (≤POD 21)
   Early postoperative complications will be classified and managed according to the Clavien-Dindo Classification (Table 8).

Table 8. Definition and grading of complication (*Clavien-Dindo* Classification)

| Grade | Definition |
|---|---|
| Grade I | Any deviation from the normal postoperative course without the need for pharmacological treatment or surgical, endoscopic, and radiological interventions Allowed therapeutic regimens for this grade: drugs as antiemetics, antipyretics, analgesics, diuretics and electrolytes, and physiotherapy. This grade also includes bedside wound manageable without local anesthetics. |
| Grade II | Requiring pharmacological treatment with drugs other than such allowed for grade I complications. This grade includes long-term fasting requiring blood transfusions and total parenteral nutrition. |
| Grade III | Requiring surgical, endoscopic, or radiological intervention |
| Grade IIIa | Intervention not under general anesthesia |
| Grade IIIb | Intervention under general anesthesia |
| Grade IV | Life-threatening complication (including CNS complications) requiring IC/ICU-management |
| Grade Iva | Single organ dysfunction (including dialysis) |
| Grade IVb | Multi-organ dysfunction |
| Grade V | Death of a patient |
| Suffix “d” | This suffix (“d” for disability) added to the respective grade of complication indicates that a follow-up is required after patient’s discharge. |

① Wound complication: anything abnormal that occurs at or around the wound site, including seroma, hematoma, wound infection, wound dehiscence, which requires intervention.
② Fluid collection/abscess: intraperitoneal fluid collection or abscess requiring intervention.
③ Intra-abdominal bleeding: bleeding through an intraperitoneal drainage tube or any bleeding confirmed by imaging that requires intervention such as blood transfusion, use of hemostatic agents, and reoperation.

④ Intraluminal bleeding: gastrointestinal bleeding including anastomotic bleeding, with symptoms such as hematemesis, anal bloody discharge, and bloody stool passage, requiring intervention such as endoscopy and blood transfusion.

⑤ Postoperative ileus: malfunction of intestinal motility causing constipation and acute abdominal pain, requiring fasting and nasogastric intubation if the condition persists until POD 5.

⑥ Anastomosis stenosis: a narrowing below the anastomotic site, which requires intervention such as fasting, drainage, endoscopy, and re-operation.

⑦ Anastomosis leakage: a clinically and radiologically confirmed leakage along the staple line.

⑧ Pancreatitis or pancreatic fistula: a pancreatitis accompanied by symptoms such as abdominal pain and fever, as confirmed by abdominal CT or elevated amylase level in the blood and abdominal drainage fluid (amylase content three times higher than serum amylase).

⑨ Pulmonary complication: postoperative atelectasis, pleural effusion, empyema, pneumonia, or pneumothorax accompanied by symptoms such as fever and shortness of breath, which requiring management with antibiotics, percutaneous drainage, or mechanical respiration (excluding breathing exercises, use of nebulizer administration, etc.)

⑩ Urinary complication: postoperative symptoms of urinary tract infection such as frequent urination, nocturia, dysuria, and other functional abnormalities of the urinary tract, requiring intervention such as curative antibiotic treatment and catheter reinsertion.

⑪ Renal complication: a postoperative renal condition requiring intervention such as medications due to elevated serum creatinine level (greater than twice the preoperative level)

⑫ Hepatic complication: a postoperative hepatic condition requiring intervention such as medications due to elevated liver function values, such as AST, ALT, and total bilirubin (more than twice the preoperative level).

⑬ Cardiac complication: an intraoperative emergency such as heart failure, myocardial ischemia, and infarction that had to be managed during surgery

⑭ Endocrine complication: postoperative endocrinal alterations related to diabetes or the adrenal gland, thyroid gland, etc.

⑮ Other: all other cases that required intervention in patients presenting with symptoms; each case must be recorded in detail.

2) Late postoperative complications (POD >21)

① Intestinal obstruction: late-onset symptoms of intestinal obstruction such as abdominal pain that begin after POD 21 or intestinal obstruction diagnosed by imaging, requiring treatment.

② Anastomotic stenosis: late-onset symptoms of anastomotic stenosis such as vomiting that begin after POD 21 and anastomotic stenosis confirmed by endoscopy, requiring treatment.

③ Chronic wound complication: chronic inflammation and hernias at the trocar insertion or incision site clinically confirmed after POD 21

④ Others: all other surgical complications that begin after POD 21, requiring treatment; each case to be recorded in detail.

**17.2 Safety evaluation of laparoscopic surgery based on early postoperative complications**

1) On POD 21 after the number of enrolled patients has reached 484, the CRO will analyze the outcomes of early complications based on the data on surgical complications and provide the analysis results to the PI within 30 days. The PI will then convene the Steering Committee within 60 days to analyze the early complication status of the operated patients and establish the non-inferiority of the laparoscopic approach by comparing the early complication rate between the laparoscopic (Arm A) and open (Arm B) surgery groups. If the non-inferiority of Arm A compared with Arm B cannot be established, the PI can discuss the possibility of discontinuing the study with the Steering Committee.
2) After the enrollment of 484 patients, further patients may be enrolled while the decision about a premature termination or continuance of the study is pending.
3) No further patients will be enrolled when the termination has been decided.
4) The rationale for setting the number of enrollments to 484 subjects is as follows.
   ① In order to ensure the safety of the subjects during the study, the time point for deciding the premature termination of the study needs to be set to discontinue the study in the event that the complication rate of Arm A (subjects undergoing laparoscopic subtotal gastrectomy with D2 lymphadenectomy) is not at least equal to that of Arm B (subjects undergoing open subtotal gastrectomy with D2 lymphadenectomy).
   ② The timing for convening the Steering Committee depends on the cumulative number of subjects in the safety analysis set (SAS). A target power is set to 90% to ensure a high test power of termination.
   ③ An interim safety analysis does not affect the efficacy such that type 1 error does not need to be adjusted.
   ④ In order to determine this timing, the cumulative number of subjects for the convening of the Steering Committee to discuss the termination was calculated as follows.
      1. As the calculation basis, the complication rate and the margin of non-inferiority for the complication rate were set to 20.9% and 12.0%, respectively, drawing on the reports by Sano et al.[21] (standard D2 gastrectomy group) and Degiuli et al.[34] (D1 gastrectomy group and D2 gastrectomy group), respectively.
      2. Accordingly, non-inferiority tests of two independent proportions (PASS 2008) were performed under the following assumptions: complication rate of both groups (Arm A and Arm B) = 20.9%, margin of non-inferiority = 12.0%, type 1 error = 0.025 (one-sided), test power = 90%.
      3. Based on the calculation results, the timing for deciding whether to terminate or continue the study was determined to be the timepoint where the number of subjects has reached 242 for each group of the SAS, totaling 484 subjects.

### 17.3 Postoperative 90-day mortality and reporting

1) Definition of postoperative 90-day mortality:
   ① All-cause death before reaching POD 90.
   ② Cases of transfer to other medical institutions, etc. with no hope for recovery also count towards 90-day mortality.

③ Death caused by early postoperative complications as listed in Section 17.1 is defined as death related to surgical complications.

2) Reporting of postoperative 90-day mortality:

① Death within POD 90 is considered a serious adverse event, and each investigator has the obligation to report it to the Coordinating PI, who will forward it to the Steering Committee (See Section 18. Adverse event reporting).

### 17.4 Adverse event assessment in patients under adjuvant chemotherapy

1) Chemotherapy-related adverse events will be assessed in patients undergoing adjuvant chemotherapy during the scheduled follow-up visits.
2) If the investigator judges that dose reduction or discontinuation of any anticancer agent is necessary, it will be executed and recorded.

## 18. Adverse event reporting

### 18.1 Serious adverse events

Any of the following events is defined as a serious adverse event (SAE):

1) Death within 90 days of surgery irrespective of its association with surgery.
2) Permanent or substantial disability (including brain lesions with lasting sequelae) with an undeniable association with surgery, regardless of duration.
3) Administration of either of the following procedures due to problems judged to have a causal relationship with early postoperative complications (to be reported in categories of definite, probable, and possible causal relationships).

① Re-operation* required at this or any other study site due to early postoperative complications**.

② Unplanned intensive care unit (ICU) care at this or any other study site due to early postoperative complications.

* Re-operation: an operation requiring general anesthesia in the operative field.

** Early complications: see Section 17.1 Methods of postoperative complication assessment and reporting

### 18.2 Reporting of serious adverse events

1) The physician in charge or the study monitor who has confirmed any SAE encountered must report it immediately to the PI. If the PI cannot be contacted, the physician in charge or the study monitor must act on his/her behalf.
2) The PI or his/her designee must complete the “SAE Report” form within 24 hours of becoming aware of the SAE and report it to the Coordinating PI (Fax.: 031-219-4468). The Coordinating PI shall report it to the Steering Committee.
3) The Steering Committee, on determining the urgency, importance, and degree of impact of the report, may notify the participating sites of the safety issue, suspending further enrollment, and discuss the termination of the study.

4) Other SAEs shall be reported to the Data and Safety Monitoring Committee and discussed in its regular meetings.

## 19 Governing bodies of the study and their roles

### 19.1 Steering Committee

1) Composition
The Steering Committee will be staffed by the Coordinating PI and four Sub-Is as the principal governing body for the conduct of the study.

2) Duration of activity
The Steering Committee shall serve throughout the study period from the application for IRB approval of the Protocol until the end of study.

3) Roles
① Discussion and decision-making regarding important study-related matters.
② Decisions on temporary suspension of patient enrollment, resumption of enrollment procedure, and termination of the study, taking into account any safety reports by the Data Safety and Monitoring Committee.
③ Decisions of termination of the study or suspension of individual investigator(s) based on the data from the interim analysis comparing the complication rate submitted by the CRO.
④ Temporary suspension of the study after notifying the participating sites of the relevant safety issue based on the decision of the urgency, importance, and degree of impact of the report received, and subsequent discussion about the termination of the study.
⑤ Attendance at the regular meetings of the Data Safety and Monitoring Committee and presentation of other SAEs for discussion.

### 19.2 Data and Safety Monitoring Committee (DSMC)

1) Composition
The Data Safety and Monitoring Committee (DSMC) is a multidisciplinary committee composed of four experts from the fields of clinical pharmacology, statistics, and surgical techniques, and two administrative officers independent of this study.

2) Duration of activity
The DSMC shall serve throughout the study period from the application of IRB approval of the Protocol until the end of study.

3) Roles
① Monitoring the subjects' safety by reviewing interim data whenever the number of patients enrolled reaches 200.
② Scope of responsibilities:
1. Safety inspection during the course of the study.
2. Regular safety assessment and handling of particular safety issues by convening emergency meetings.

3. Advice on the necessity of Protocol modification/revision to minimize the subjects' risk.
4. Request for additional information or provision of recommendations including suspension of enrollment and termination of study if necessary.

③ The Chairperson of the DSMC is responsible for providing the investigators with a summary/discussion details of the regular meetings and the minutes with dates and attendees specified, and for documenting all study-related correspondence with opinions converged among the DSMC members.

### 19.3 Advisory Committee

① The Advisory Committee shall hold ad hoc meetings to discuss matters regarding the drafting and implementing the study protocol and constitute an advisory body to support the Steering Committee.

② The Advisory Committee is composed of four persons independent of the study.

### 19.4 Clinical Research Organization

① Overall administration of the conduct of the study.

② Study site monitoring.

③ eCRFs and database management.

## 20. Study outcome database management

### 20.1 Study site monitoring

① The CRO will review the study protocol and eCRFs together with the PI(s) and Sub-I(s) in the initiation visit at the study site or at the Investigator Meeting prior to the commencement of the study. Throughout the study duration, CRO monitors will regularly visit their respective participating sites to check the completeness of the patient records, the accuracy of the eCRFs, and the progress status of the Protocol and enrollment, and to inspect whether the conduct of the study is in compliance with the pertinent regulations and guidelines. Investigators must provide maximum assistance to the CRO monitors during these monitoring sessions.

② The investigator keeps the patient demographics, medical information, lab test data, and source documents containing other test and assessment results of each subject. All information included in the eCRFs will be rendered traceable based on the source documents kept in each subject's folder. The investigator will keep one original copy of the ICF signed and dated by the subject and provides one copy to the subject.

③ The investigator must allow the CRO monitor to view the related source documents to audit the contents of eCRFs against the records of the source documents. The monitoring will be carried out according to the following criteria: the existence of signed and dated ICF, compliance with inclusion/exclusion criteria, recording of SAEs, and standard validation of all data to be used as efficacy and safety outcome measures. Additional confirmation of the

consistency of source documents and eCRFs may be performed in accordance with the specific monitoring plan of the study. No information will be disclosed about the subject's identity on the source documents.

**20.2 Case report and database management**

① The CRO monitor can inspect the completeness and accuracy of the eCRFs drafted by the site monitor (Sub-I), and request necessary modifications and additions of data to the site monitor. Obvious data errors must be corrected by the data manager and a Data Clarification Form (DCF) will be issued to the study site for the data requiring confirmation. The site monitor will modify the related data and send the revised data back to the data manager.

② On completion of the data management quality control procedure, the data will be stored as frozen data. The original copy of the eCRFs will be kept by the Coordinating PI, and a copy will be kept by the PIs of the participating sites.

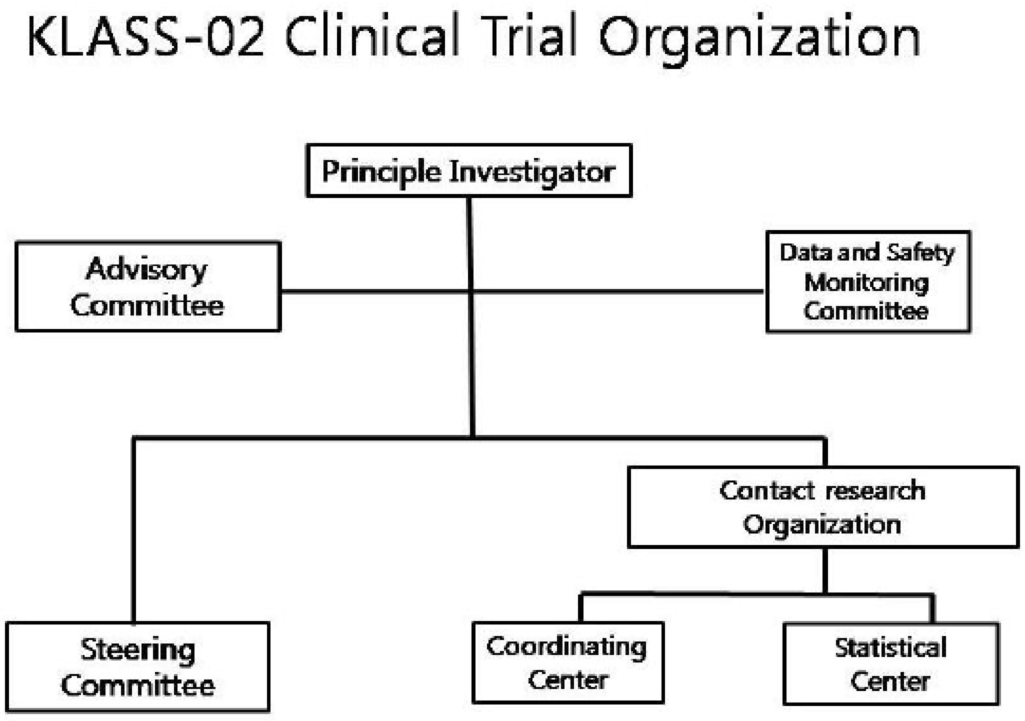


**21. Subject information sheet and informed consent form**

1) Prior to the conduct of this study, the investigator (PI and Sub-I) must obtain a signed and dated ICF from each subject (or legal representative) after sufficiently explaining the contents of the study, the effects of the surgical procedures, and anticipated adverse events.

2) The investigator will keep the original signed and dated ICF in the study folder, and a copy of the signed and dated ICF will be provided to the subject (or legal representative) along with the SIS.

## 22. Additional use of data

1) The study-related data can be used during the course or completion of the study for purposes other than the conduct of the study subject to approval by the Steering Committee.
2) Use of data for other purposes is limited to the date related to the subjects who signed a separate ICF allowing the use of data for other purposes.
3) Use of data for other purposes requires prior approval by the IRB of the medical institution of the participating site concerned.
4) The document retention period for the study data whose use has been approved the IRB is 15 years from the day of study end.

## 23. Study-related data and document archiving

### 23.1 Data management

1) Study-related documents include enrollment logs, delegation logs, source documents, monitoring files, appointment schedules, correspondence between the sponsor and the investigator, and regulatory files, for example, the Protocol signed by the investigator and all its revised versions, IRB-related letters, approval-related files, IRB-approved ICFs signed and dated by the subjects, site drug accountability log, etc.
2) Source documents include all observations (measurements and assessments) and records of clinical study activities and all reports and records necessary for the evaluation and reproduction of the study. Therefore, the source documents should include records of all treatments per protocol or equivalent treatments.
3) Observations should be kept as source documents in original copies as far as possible.
4) However, if a photocopy is available which is exactly the same as the original, clean, and easy to read, it can also be regarded as the original source document.
5) Pursuant to the regulatory standards and requirements of the government bodies, all documents related to the conduct of a clinical study must be kept by the investigator/Head of the study site and the Sponsor.
   ① In compliance with Article 32 of the Enforcement Regulations of the Pharmaceutical Affairs Act (Ministry of Health and Welfare, Decree No. 122, 01Jul2009), these documents should be kept for three years from the completion date of the clinical study.
   ② However, if ordered by the Minister of Food and Drug Safety or deemed necessary by the Sponsor, the retention period should be extended.

③ The document retention period for the study data, whose use for purposes other than the study has been approved by the IRB and permitted by the subject, is 15 years from the day of study end.

### 23.2 Confidentiality

1) Particular care should be taken to ensure that all records identifying the patient be kept confidential.
2) However, CRO monitors, auditors, IRB members, and the Minister of Food and Drug Safety may view the subject records in order to verify the reliability of the study procedures and study-related data within the scope of the related regulations without infringing on the confidentiality of the subjects.

### 23.3 Access to the study-related data and literature

The investigator and the study site will make all study-related data and literature available for viewing for the purpose of review by CRO monitors, auditors, IRB members, and government survey.

### 23.4 Quality control (QC) and quality assurance (QA) of study data

1) Data obtained during the course of the study should be entered directly into the eCRF by the investigator.
2) The CRO monitor can check the entered eCRF records against the source documents to ensure accuracy and request modifications and addition of data to the investigator if deemed necessary.
3) Obvious data errors will be corrected by the data manager and the Data Clarification Form (DCF) will be issued for data requiring confirmation to request the study site to modify the data and send the revised data back to the data manager.
4) To ensure the reliability of the study, all important observations (measurement and assessment) items related to the study should be documented and archived for three years from the date of study end together with the essential study documents.

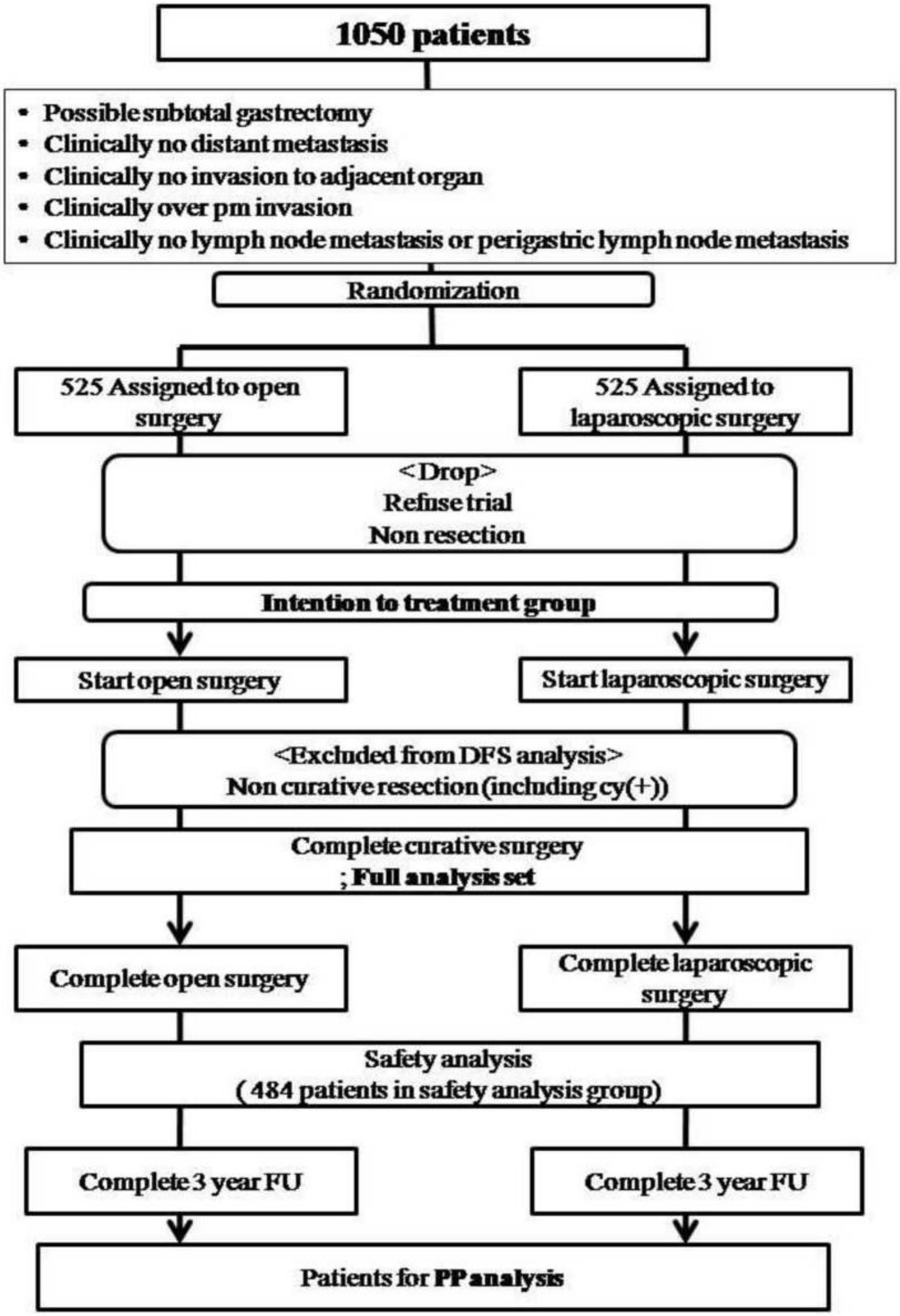


Fig. Study design schematic and patient disposition

(FU: follow up, PP: per protocol analysis)

## 24. References

1. Ohgami M, Otani Y, Kumai K, Kubota T, Kim YI, Kitajima M. Curative laparoscopic surgery for early gastric cancer: five years experience. *World J Surg.* 1999;23(2):187-192; discussion 192-183.
2. Kitano S, Iso Y, Moriyama M, Sigimachi K. Laparoscopy-assisted Billroth I gastrectomy. *Surg Laparosc Endosc.* 1994;4(2):146-148.
3. Choi SH, Yoon DS, Chi HS, Min JS. Laparoscopy-assisted radical subtotal gastrectomy for early gastric carcinoma. *Yonsei Med J.* 1996;37(3):174-180.
4. Kim MC, Kim W, Kim HH, et al. Risk factors associated with complication following laparoscopy-assisted gastrectomy for gastric cancer: a large-scale Korean multicenter study. *Ann Surg Oncol.* 2008;15(10):2692-2700.
5. Kim W, Song KY, Lee HJ, Han SU, Hyung WJ, Cho GS. The impact of comorbidity on surgical outcomes in laparoscopy-assisted distal gastrectomy: a retrospective analysis of multicenter results. *Ann Surg.* 2008;248(5):793- 799.
6. Kitano S, Shiraishi N, Uyama I, Sugihara K, Tanigawa N. A multicenter study on oncologic outcome of laparoscopic gastrectomy for early cancer in Japan. *Ann Surg.* 2007;245(1):68-72.
7. Hayashi H, Ochiai T, Shimada H, Gunji Y. Prospective randomized study of open versus laparoscopy-assisted distal gastrectomy with extraperigastric lymph node dissection for early gastric cancer. *Surg Endosc.* 2005;19(9):1172-1176.
8. Huscher CG, Mingoli A, Sgarzini G, et al. Laparoscopic versus open subtotal gastrectomy for distal gastric cancer: five-year results of a randomized prospective trial. *Ann Surg.* 2005;241(2):232-237.

9. Kitano S, Shiraishi N, Fujii K, Yasuda K, Inomata M, Adachi Y. A randomized controlled trial comparing open vs laparoscopy-assisted distal gastrectomy for the treatment of early gastric cancer： an interim report. *Surgery.* 2002;131(1 Suppl):S306-311.

10. Lee JH, Han HS. A prospective randomized study comparing open vs laparoscopy-assisted distal gastrectomy in early gastric cancer: early results. *Sug Endosc.* 2005;19(2):168-173.

11. Kim HH, Hyung WJ, Cho GS, et al. Morbidity and mortality of laparoscopic gastrectomy versus open gastrectomy for gastric cancer: an interim report–a phase III multicenter, prospective, randomized Trial (KLASS Trial). *Ann Sug.* ;251(3):417-420.

12. Bonenkamp JJ, Hermans J, Sasako M, et al. Extended lymph-node dissection for gastric cancer. *N Engl J Med.* 1999;340(1 2):908-914.

13. Bonenkamp JJ, Songun I, Hermans J, et al. Randomised comparison of morbidity after D1 and D2 dissection for gastric cancer in 996 Dutch patients. *Lancet.* 1995;345(8952):745-748.

14. Cuschieri A, Fayers P, Fielding J, et al. Postoperative morbidity and mortality after D1 and D2 resections for gastric cancer: preliminary results of the MRC randomised controlled surgical trial. The Surgical Cooperative Group. *Lancet.* 1996;347(9007):995-999.

15. Cuschieri A, Weeden S, Fielding J, et al. Patient survival after D1 and D2 resections for gastric cancer: long-term results of the MRC randomized surgical trial. Surgical Co-operative Group. *Br J Cancer.* 1999;79(9-10):1522-1530.

16. Sasako M. Clinical trials of surgical treatment of malignant diseases. *Int J Clin Oncol.* 2005;10(3):165-170.

17. Maruyama K, Okabayashi K, Kinoshita T. Progress in gastric cancer surgery in Japan and its limits of radicality. *World J Sug.* 1987;11(4):418-425.

18. Sawai K, Takahashi T, Suzuki H. New trends in surgery for gastric cancer in Japan. *J Surg Oncol.* 1994;56(4):221-226.

19. Kodera Y, Sasako M, Yamamoto S, Sano T, Nashimoto A, Kurita A. Identification of risk factors for the development of complications following extended and superextended lymphadenectomies for gastric cancer. *Br J Surg.* 2005;92(9):1103-1109.

20. Kulig J, Popiela T, Kolodziejczyk P, Sierzega M, Szczepanik A. Standard D2 versus extended D2 (D2+) lymphadenectomy for gastric cancer: an interim safety analysis of a multicenter, randomized, clinical trial. *Am J Surg.* 2007;193(1):10-15.

21. Sano T, Sasako M, Yamamoto S, et al. Gastric cancer surgery: morbidity and mortality results from a prospective randomized controlled trial comparing D2 and extended para-aortic lymphadenectomy--Japan Clinical Oncology Group study 9501. *J Cln Oncol* 2004;22(14):2767-2773.

22. Sasako M, Sano T, Yamamoto S, et al. D2 lymphadenectomy alone or with paraaortic nodal dissection for gastric cancer. *N Engl J Med.* 2008;359(5):453-462.

23. Nakajima T. Gastric cancer treatment guidelines in Japan. *Gastric Cancer.* 2002;5(1):1-5.

24. Kawamura H, Okada K, Isizu H, et al. Laparoscopic gastrectomy for early gastric cancer targeting as a less invasive procedure. *Surg Endosc.* 2008;22(1):81 - 85.

25. Song KY, Kim SN, Park CH. Laparoscopy-assisted distal gastrectomy with D2 lymph node dissection for gastric cancer: technical and oncologic aspects. *Surg Endosc.* 2008;22(3):655-659.

26. Tokunaga M, Hiki N, Fukunaga T, et al. Laparoscopy-assisted distal gastrectomy with D2 lymph node dissection following standardization–a preliminary study. *J Gastrointest Surg.* 2009;13(6):1058-1063.

27. Huang JL, Wei HB, Zheng ZH, et al. Laparoscopy-assisted D2 radical distal gastrectomy for advanced gastric cancer. *Dig Surg.* 2010;27(4):291-296.

28. Hur H, Jeon HM, Kim W. Laparoscopy-assisted distal gastrectomy with D2 lymphadenectomy for T2b advanced gastric cancers: three years' experience. *J Surg Oncol.* 2008;98(7):515-519.

29. Hwang SI, Kim HO, Yoo CH, Shin JH, Son BH. Laparoscopic-assisted distal gastrectomy versus open distal gastrectomy for advanced gastric cancer. *Surg Endosc.* 2009;23(6):1252-1258.

30. Lee J, Kim W. Long-term outcomes after laparoscopy-assisted gastrectomy for advanced gastric cancer: analysis of consecutive 106 experiences. *J Surg Oncol.* 2009;100(8):693-698.

31. Sakuramoto S, Sasako M, Yamaguchi T, et al. Adjuvant chemotherapy for gastric cancer with S-1, an oral fluoropyrimidine. *N Engl J Med.* 2007;357(18):1810-1820.

32. Jung SH, Kang SJ, McCall LM, Blumenstein B. Sample size computation for two-sample noninferiority log-rank test. *J Biopharm Stat.* 2005;15(6):969-979.

33. Freidlin B, Korn EL. Stopping clinical trials early for benefit: impact on estimation. *CHn Trials.* 2009;6(2):119-125.

34. Degiuli M, Sasako M, Ponti A. Morbidity and mortality in the Italian Gastric Cancer Study Group randomized clinical trial of D1 versus D2 resection for gastric cancer. *Br J Surg.* 1997(5):643-649.
