# Supplementary material for: Laparoscopic vs Open Distal Gastrectomy for Locally Advanced Gastric Cancer: 5-Year Outcomes of the KLASS-02 Randomized Clinical Trial
Source: JAMA Surg. 2022 Jul 20;157(10):879–86. doi: 10.1001/jamasurg.2022.2749 (PMC9301593; doi:10.1001/jamasurg.2022.2749)
Supplement: Supplement 2. — eFigure 1. Trial Profile eFigure 2. Kaplan-Meier Analyses of Relapse-Free Survival Following Laparoscopic Gastrectomy and Open Gastrectomy (cStage FAS Data Set) eFigure 3. Kaplan-Meier Analyses of Overall Survival Following Laparoscopic Gastrectomy and Open Gastrectomy (pStage FAS Data Set) eFigure 4. Kaplan-Meier Analyses of Relapse-Free Survival Following Laparoscopic Gastrectomy and Open Gastrectomy (pStage FAS Data Set) eFigure 5. Kaplan-Meier Analyses of Overall Survival Following Laparoscopic Gastrectomy and Open Gastrectomy (cStage ITT Data Set) eFigure 6. Kaplan-Meier Analyses of Overall Survival Following Laparoscopic Gastrectomy and Open Gastrectomy (pStage ITT Data Set) eFigure 7. Kaplan-Meier Analyses of Overall Survival Following Laparoscopic Gastrectomy and Open Gastrectomy in Patients Who Completed Adjuvant Chemotherapy (pStage FAS Data Set) eFigure 8. Recurrence Patterns in Patients Who Underwent Laparoscopic Gastrectomy and Open Gastrectomy eTable 1. Patient Clinicopathological Characteristics eTable 2. Distribution of Long-term Surgical Complications (>21 Postoperative Days) in Patients Who Underwent Laparoscopic Gastrectomy and Open Gastrectomy [file jamasurg-e222749-s002.pdf]

Supplementary Online Content

Son SY, Hur H, Hyung WJ, et al; Korean Laparoendoscopic Gastrointestinal Surgery Study (KLASS) Group. Laparoscopic vs open distal gastrectomy for locally advanced gastric cancer: 5-year outcomes of the KLASS-02 randomized clinical trial. *JAMA Surg*. Published online July 20, 2022. doi:10.1001/jamasurg.2022.2749

eFigure 1. Trial Profile

eFigure 2. Kaplan-Meier Analyses of Relapse-Free Survival Following Laparoscopic Gastrectomy and Open Gastrectomy (cStage FAS Data Set)

eFigure 3. Kaplan-Meier Analyses of Overall Survival Following Laparoscopic Gastrectomy and Open Gastrectomy (pStage FAS Data Set)

eFigure 4. Kaplan-Meier Analyses of Relapse-Free Survival Following Laparoscopic Gastrectomy and Open Gastrectomy (pStage FAS Data Set)

eFigure 5. Kaplan-Meier Analyses of Overall Survival Following Laparoscopic Gastrectomy and Open Gastrectomy (cStage ITT Data Set)

eFigure 6. Kaplan-Meier Analyses of Overall Survival Following Laparoscopic Gastrectomy and Open Gastrectomy (pStage ITT Data Set)

eFigure 7. Kaplan-Meier Analyses of Overall Survival Following Laparoscopic Gastrectomy and Open Gastrectomy in Patients Who Completed Adjuvant Chemotherapy (pStage FAS Data Set)

eFigure 8. Recurrence Patterns in Patients Who Underwent Laparoscopic Gastrectomy and Open Gastrectomy

eTable 1. Patient Clinicopathological Characteristics

eTable 2. Distribution of Long-term Surgical Complications (>21 Postoperative Days) in Patients Who Underwent Laparoscopic Gastrectomy and Open Gastrectomy

This supplementary material has been provided by the authors to give readers additional information about their work.

© 2022 Son SY et al. *JAMA Surgery.*

eFigure 1. Trial Profile

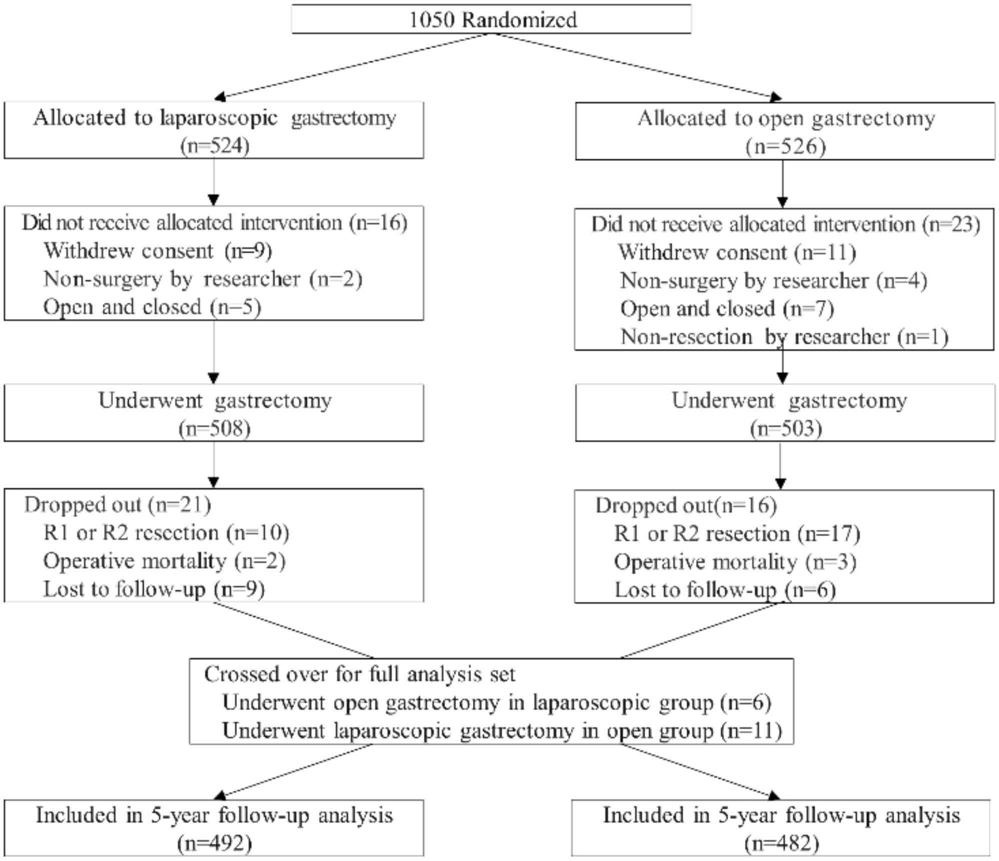

© 2022 Son SY et al. *JAMA Surgery.*

eFigure 2. Kaplan-Meier Analyses of Relapse-Free Survival Following Laparoscopic Gastrectomy and Open Gastrectomy in (A) all patients and patients with (B) cStage I, (C) cStage II, (D) and cStage III gastric cancer, based on the 8th TNM staging system [the FAS data set].

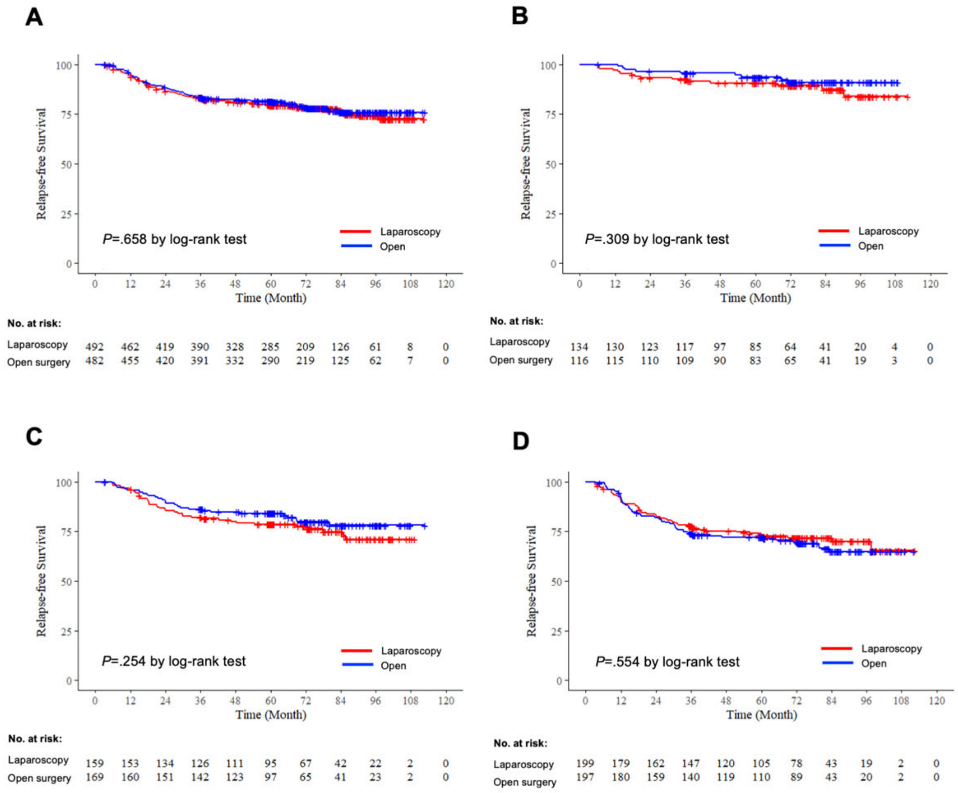

© 2022 Son SY et al. *JAMA Surgery.*

eFigure 3. Kaplan-Meier Analyses of Overall Survival Following Laparoscopic Gastrectomy and Open Gastrectomy in (A) all patients and patients with (B) pStage I, (C) pStage II, (D) and pStage III gastric cancer, based on the 8th TNM staging system [the FAS dataset].

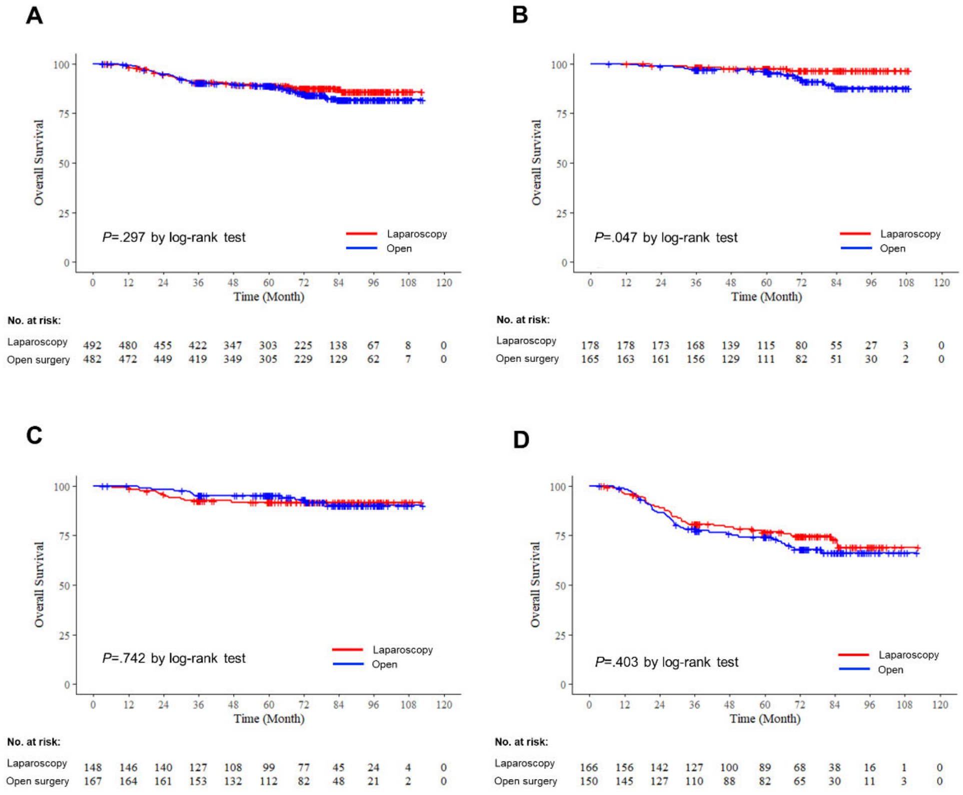

© 2022 Son SY et al. *JAMA Surgery.*

eFigure 4. Kaplan-Meier Analyses of Relapse-Free Survival Following Laparoscopic Gastrectomy and Open Gastrectomy in (A) all patients and patients with (B) pStage I, (C) pStage II, (D) and pStage III gastric cancer, based on the 8th TNM staging system [the FAS dataset].

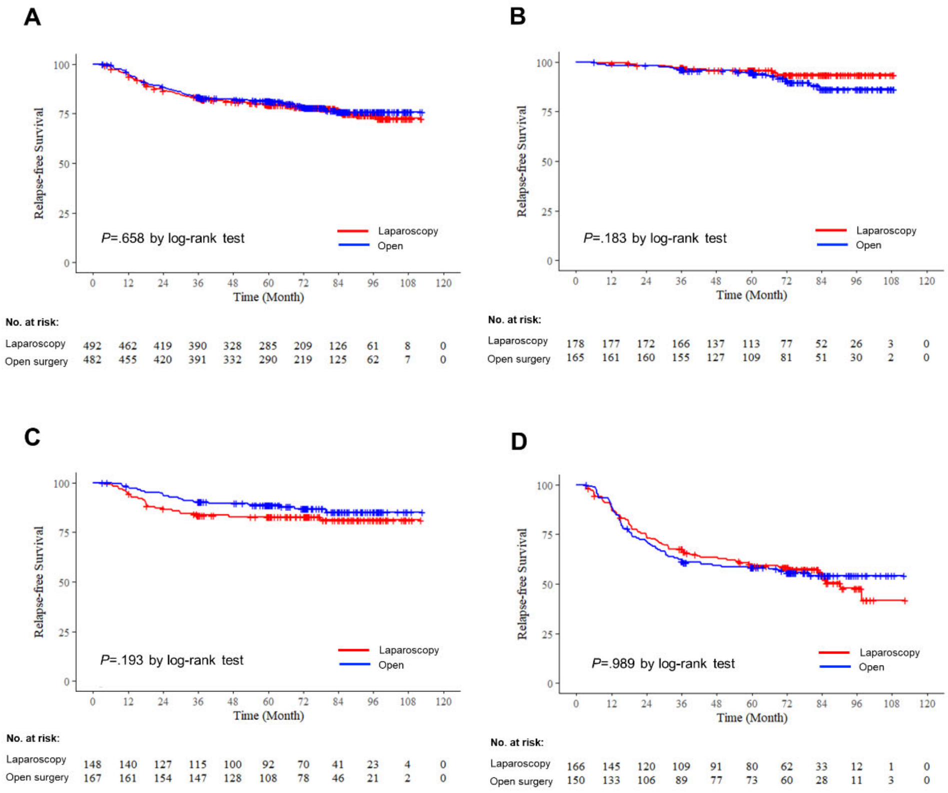

© 2022 Son SY et al. *JAMA Surgery.*

eFigure 5. Kaplan-Meier Analyses of Overall Survival Following Laparoscopic Gastrectomy and Open Gastrectomy in (A) all patients and patients with (B) cStage I, (C) cStage II, (D) and cStage III gastric cancer, based on the 8th TNM staging system [the ITT data set].

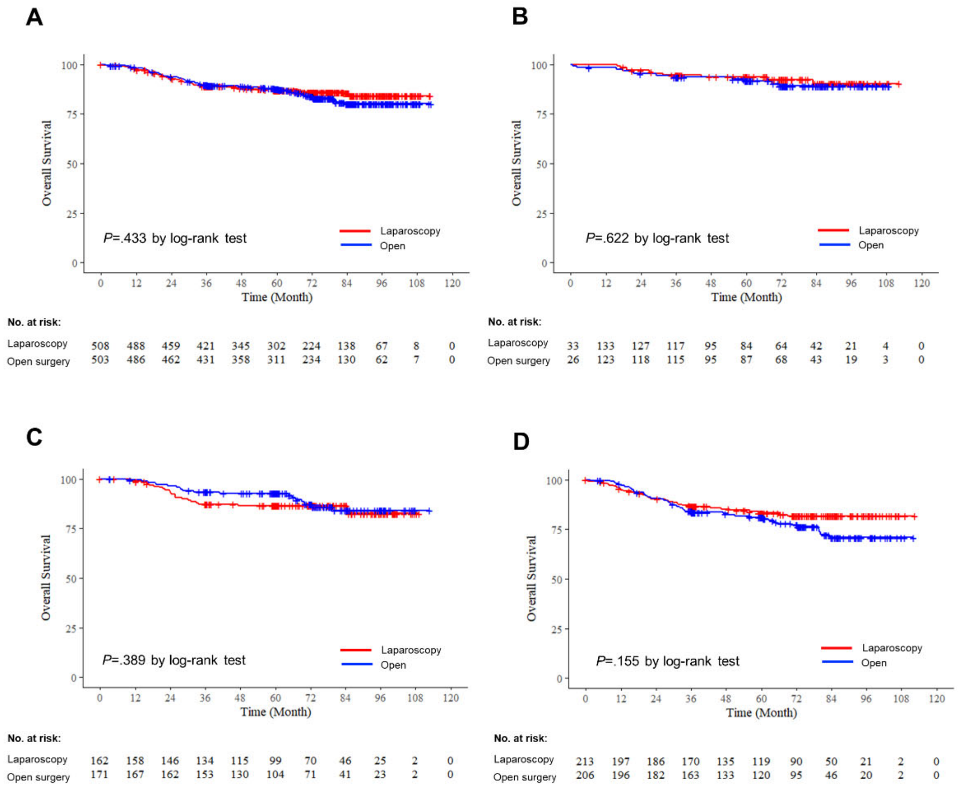

© 2022 Son SY et al. *JAMA Surgery.*

eFigure 6. Kaplan-Meier Analyses of Overall Survival Following Laparoscopic Gastrectomy and Open Gastrectomy in (A) all patients and patients with (B) pStage I, (C) pStage II, (D) and pStage III gastric cancer, based on the 8th TNM staging system [the ITT data set].

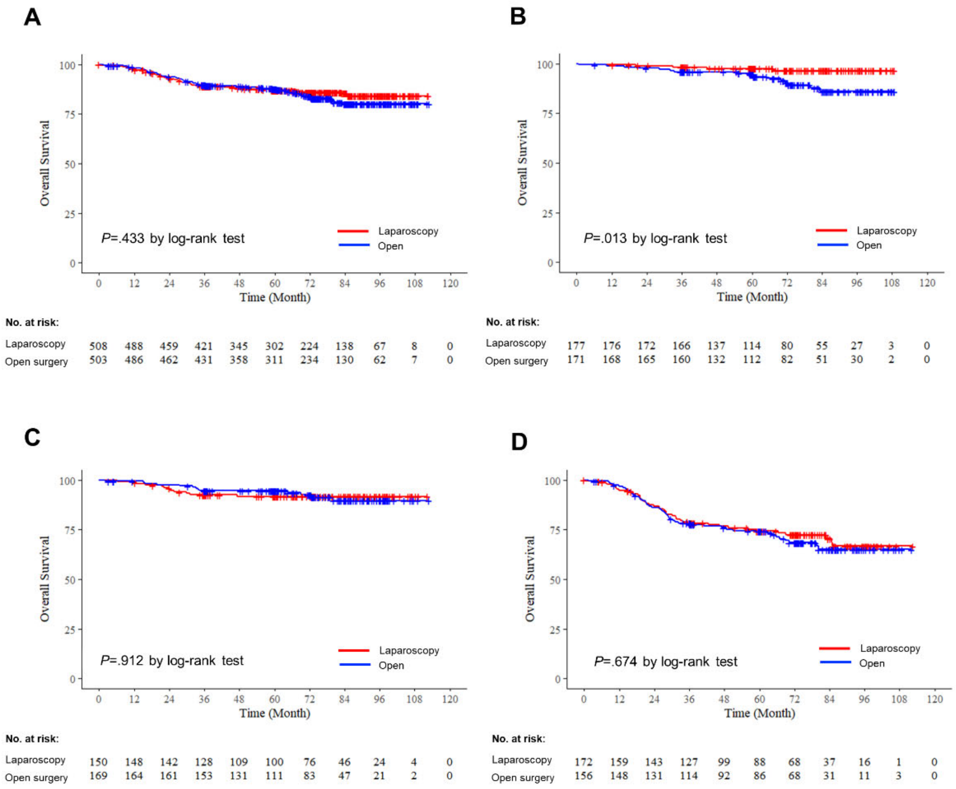

© 2022 Son SY et al. *JAMA Surgery.*

eFigure 7. Kaplan-Meier Analyses of Overall Survival Following Laparoscopic Gastrectomy and Open Gastrectomy in Patients Who Completed Adjuvant Chemotherapy (pStage FAS Data Set)

(A) all patients and patients with (B) pStage I, (C) pStage II, (D) and pStage III gastric cancer, based on the 8th TNM staging system [the FAS Data Set]

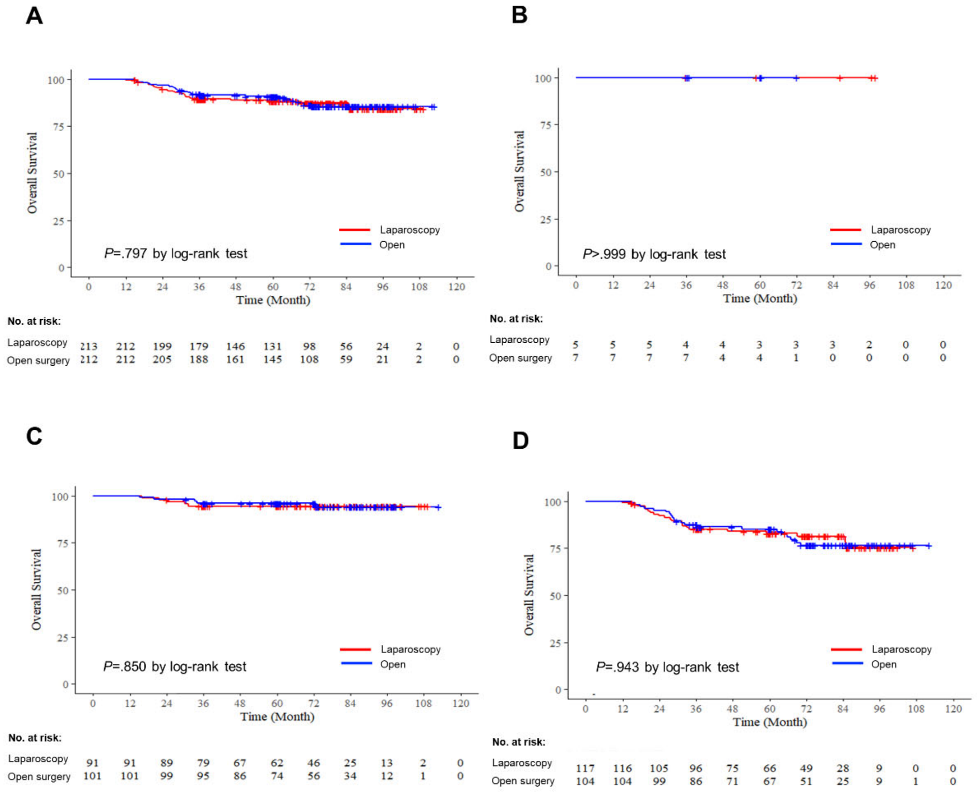

© 2022 Son SY et al. *JAMA Surgery.*

eFigure 8. Recurrence Patterns in Patients Who Underwent Laparoscopic Gastrectomy and Open Gastrectomy

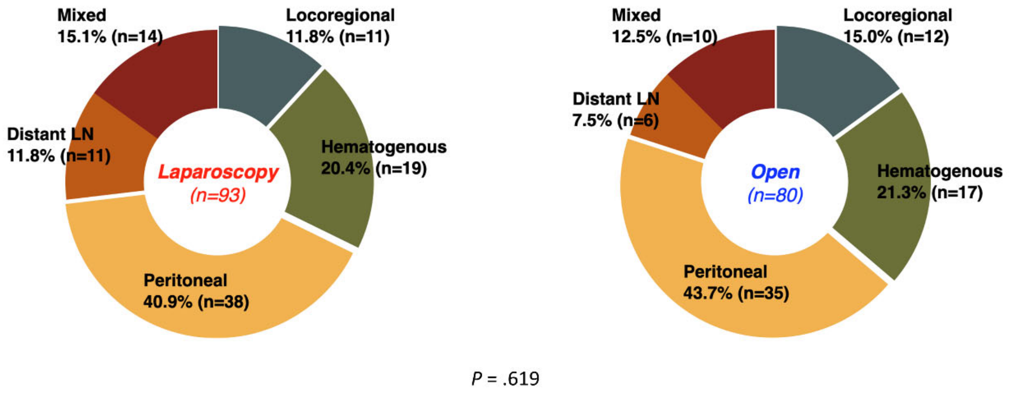

© 2022 Son SY et al. *JAMA Surgery.*

eTable 1. Patient Clinicopathological CharacteristiScs [the ITT dataset]

Variable	Laparoscopy (n=508)	Open (n=503)	p value
Age (years)	59.8 ± 11.0	59.5 ± 11.6	.664
Sex, n (%)			.269
Men	368 (72.4)	348 (69.2)	
Women	140 (27.6)	155 (30.8)	
BMI (kg/m^2)	23.5 ± 3.0	23.7 ± 3.2	.306
ASA group, n (%)			.612
I	243 (47.8)	246 (48.9)	
II	237 (46.7)	236 (46.9)	
III	28 (5.5)	21 (4.2)	
Extent of resection, n (%)			.724
Distal gastrectomy	490 (96.5)	488 (97.0)	
Total gastrectomy	18 (3.5)	15 (3.0)	
Extent of lymphadenectomy, n (%)			.450
< D2	2 (0.4)	4 (0.8)	
D2	506 (99.6)	499 (99.2)	
Tumor size (cm)	4.6 ± 2.5	4.6 ± 2.4	.652
Retrieved lymph nodes, n (%)	46.9 ± 18.0	47.1 ± 16.4	.812
Metastatic lymph nodes	3.8 ± 6.3	3.8 ± 6.7	.976
Histology, n (%)			.389
Differentiated	203 (40.0)	197 (39.2)	
Undifferentiated	295 (58.1)	289 (57.5)	
Others	10 (2.0)	17 (3.4)	
Pathological T classification, n (%)			.602
T1	138 (27.2)	129 (25.6)	
T2	101 (19.9)	117 (23.3)	
T3	138 (27.2)	136 (27.0)	
T4	131 (25.8)	121 (24.1)	
Pathological N classification, n (%)			.899
N0	224 (44.1)	224 (44.5)	
N+	284 (55.9)	279 (55.5)	
Pathological 8th TNM stage, n (%)			.529
I	177 (34.8)	171 (34.0)	
II	150 (29.5)	169 (33.6)	
III	172 (33.9)	156 (31.0)	
Ⅳ	9 (1.8)	7 (1.4)	
Neoadjuvant chemotherapy	0 (0.0)	0 (0.0)	
Postoperative adjuvant chemotherapy, n (%)			
Received	305 (60.0)	309 (61.4)	.653
TS-1	163 (53.4)	188 (60.8)	.316

© 2022 Son SY et al. *JAMA Surgery.*

XELOX (Xeloda)	100 (32.8)	85 (27.5)	
Fluorouridine	19 (6.2)	15 (4.9)	
Other	23 (7.5)	21 (6.8)	
Completed	212 (75.2)	216 (75.0)	>.999
Dose reduction	103 (33.8)	106 (34.3)	.932
Time interval to adjuvant chemotherapy (weeks)	4.9 ± 2.0	5.1 ± 1.7	.202

Results expressed as mean ± standard deviation (SD) or as n (%)

BMI, body mass Index; ASA, American Society of Anesthesiologists

© 2022 Son SY et al. *JAMA Surgery.*

eTable 2. Distribution of Long-term Surgical Complications (>21 Postoperative Days) in Patients Who Underwent Laparoscopic Gastrectomy and Open Gastrectomy

Variable	Laparoscopy (n=492)	Open (n=482)	p value†
Follow-up period (months)*	68.0 (4.1–112.7)	70.1 (3.3–112.9)	
Total late complication (%)	32 (6.5)	53 (11.0)	.011
Intestinal obstruction	13 (2.6)	24 (5.0)	.056
Stenosis	0	0	
Fluid collection or abscess	3 (0.6)	2 (0.4)	.671
Delayed gastric emptying	1 (0.2)	4 (0.8)	.171
Reflux symptom	0	3 (0.6)	.082
Post-gastrectomy symptoms	3 (0.6)	3 (0.6)	.983
Chronic wound complication	3 (0.6)	9 (1.9)	.077
Incisional hernia	2 (0.4)	7 (1.4)	
Others	11 (2.2)	12 (2.5)	.803
Clavien-Dindo classification (%)			
Grade I or II	18 (3.7)	32 (6.6)	.359
Grade III or higher	14 (2.8)	21 (4.4)	.777

* Median (range), † p values were determined by log-rank tests

© 2022 Son SY et al. *JAMA Surgery.*
