# Supplementary material for: Laparoscopic vs Open Distal Gastrectomy for Locally Advanced Gastric Cancer: 5-Year Outcomes of the KLASS-02 Randomized Clinical Trial
Source: JAMA Surg. 2022 Jul 20;157(10):879–86. doi: 10.1001/jamasurg.2022.2749 (PMC9301593; doi:10.1001/jamasurg.2022.2749)
Supplement: Supplement 3. — Nonauthor Collaborators [file jamasurg-e222749-s003.pdf]

Supplemental Online Content: Nonauthor Collaborators

*Indicates required information. Only first name, last name, and suffix will appear in PubMed.

*Group Name(s): Korean Laparoendoscopic Gastrointestinal Surgery Study (KLASS) Group

***First Name and Middle Initial(s)**	***Last Name**	***Suffix (eg, Jr, III)**	Academic Degrees	Institution	Location (city, state/province, country)	Role or Contribution, eg, chair, principal investigator	Group (if more than 1 Group listed in the byline) and/or Subgroup (eg, Steering Committee)
HyeSeong	Ahn		MD	Seoul National University			
Sang-Hoon	Ahn		MD	Seoul National University			
Jae-Moon	Bae		MD	Sungkyunkwan University			
Jung Min	Bae		MD	Yeungnam University			
Sung Joon	Bae		MD	Korea Institute of Radiological & Medical Sciences			
Cheul Su	Byun		MD	Dongsuwon Hospital			
Yeon Soo	Chang		MD	Eulji University			
Hyunjin	Cho		MD	Inje University			
In	Cho		MD	Soonchunhyang University			
Minah	Cho		MD	Yonsei University			
Chang In	Choi		MD	Pusan National University			
Kyeong-Woon	Choi		MD	Inje University			
Seohee	Choi		MD	National Health Insurance Service Ilsan Hospital			
Seung-Ho	Choi		MD	Yonsei University			
Sung Il	Choi		MD	Kyung Hee University			
Yun-Suck	Choi		MD	Inha University			
Ho-Young	Chung		MD	Kyungpook National University			
Joo Won	Chung		MD	Kosin University			
Jung Kee	Chung		MD	Seoul National University			
Bang Wool	Eom		MD	National Cancer Center			
Sang Soo	Eom		MD	National Cancer Center			
Dongyeop	Ha		MD	Pohan St. Mary's Hospital			
Man Ho	Ha		MD	Kwangdong University International St.Mary's Hospital			
Taekyung	Ha		MD	Hanyang Unversity			
Dong-Seok	Han		MD	Seoul National University			
Sang-Moon	Han		MD	Seoul Medical Center			

Supplemental Online Content: Nonauthor Collaborators

*Indicates required information. Only first name, last name, and suffix will appear in PubMed.

*First Name and Middle Initial(s)	*Last Name	*Suffix (eg, Jr, III)	Academic Degrees	Institution	Location (city, state/province, country)	Role or Contribution, eg, chair, principal investigator	Group (if more than 1 Group listed in the byline) and/or Subgroup (eg, Steering Committee)
Se Wung	Han		MD	Chonbuk National University			
Yeon-Ju	Huh		MD	Seoul National University			
Sun-Hwi	Hwang		MD	Pusan National University			
You-Jin	Jang		MD	Korea University			
Ye Seob	Jee		MD	Dankook Unversity			
Chul Hyo	Jeon		MD	The Catholic University of Korea			
Gui-Ae	Jeong		MD	Soonchunhyang University			
Inho	Jeong		MD	Jeju National University			
Oh	Jeong		MD	Chonnam National Univeristy			
Sang-Ho	Jeong		MD	Gyeongsang National University			
Sung-Ho	Jin		MD	Korea Institute of Radiological & Medical Sciences			
Ji Hoon	Jo		MD	Kosin University			
Kyong Hwa	Jun		MD	The Catholic University of Korea			
Bong Soo	Jung		MD	Andong Hospital			
Do Hyun	Jung		MD	Young Do Hospital			
Mi-Ran	Jung		MD	Chonnam National Univeristy			
Yoonju	Jung		MD	The Catholic University of Korea			
Dongbaek	Kang		MD	Wongkwang University			
Ji Hoon	Kang		MD	Chonnam National Univeristy			
So Hyun	Kang		MD	Seoul National University			
Amy	Kim		MD	University of Ulsan			
Beom Su	Kim		MD	University of Ulsan			
Chan Young	Kim		MD	Chonbuk National University			
Chang Hyun	Kim		MD	SAM Hospital			
Chee Young	Kim		MD	Busan Hangun Hospital Bumcheon campus			

Supplemental Online Content: Nonauthor Collaborators

*Indicates required information. Only first name, last name, and suffix will appear in PubMed.

***First Name and Middle Initial(s)**	***Last Name**	***Suffix (eg, Jr, III)**	Academic Degrees	Institution	Location (city, state/province, country)	Role or Contribution, eg, chair, principal investigator	Group (if more than 1 Group listed in the byline) and/or Subgroup (eg, Steering Committee)
Dae Hoon	Kim		MD	Chungbuk National University			
Dong Jin	Kim		MD	The Catholic University of Korea			
Dong -Wook	Kim		MD	Dankook Unversity			
Eun Young	Kim		MD	The Catholic University of Korea			
Ho-Goon	Kim		MD	Chonnam National Univeristy			
Hyun Il	Kim		MD	Hanyang Unversity			
In Ho	Kim		MD	Daegu Catholic University			
Ji Hoon	Kim		MD	University of Ulsan			
Jong-Han	Kim		MD	Korea University			
Jun Young	Kim		MD	Shintong Hospital			
Kap-Jung	Kim		MD	Hallym University			
Ki Han	Kim		MD	Dong-A University			
Kwang Hee	Kim		MD	Inje University			
Kyoung Hwan	Kim		MD	Cheju Halla Hospital			
Min Gyu	Kim		MD	Hanyang Unversity			
Sa-Hong	Kim		MD	Seoul National University			
Sang Woon	Kim		MD	Yeungnam University			
Se Won	Kim		MD	Dong Kang Medical Center			
Seong-Gon	Kim		MD	Konyang University			
Su Mi	Kim		MD	CHA University			
Sung	Kim		MD	Sungkyunkwan University			
Sung Geun	Kim		MD	The Catholic University of Korea			
Sung Soo	Kim		MD	Jeju National University			
Tae Han	Kim		MD	Gyeongsang National University			
Yong Ho	Kim		MD	Kyung Hee University			
Yongil	Kim		MD	Ewha Womans University			
Yoo Min	Kim		MD	Yonsei University			

Supplemental Online Content: Nonauthor Collaborators

*Indicates required information. Only first name, last name, and suffix will appear in PubMed.

*First Name and Middle Initial(s)	*Last Name	*Suffix (eg, Jr, III)	Academic Degrees	Institution	Location (city, state/province, country)	Role or Contribution, eg, chair, principal investigator	Group (if more than 1 Group listed in the byline) and/or Subgroup (eg, Steering Committee)
You Na	Kim		MD	Ewha Womans University			
Jeong Hyun	Ko		MD	Saegyaero Hospital			
Sung Chan	Kong		MD	Yonsei University			
In Gyu	Kwon		MD	Yonsei University			
Ji Hyoung	Kwon		MD	Gyurim Geriatric Hospital			
Oh Kyoung	Kwon		MD	Kyungpook National University			
Chang Min	Lee		MD	Korea University			
Han Hong	Lee		MD	The Catholic University of Korea			
Joong Ho	Lee		MD	Yonsei University			
Jun Ho	Lee		MD	Sungkyunkwan University			
Jun Hyun	Lee		MD	The Catholic University of Korea			
In Seob	Lee		MD	University of Ulsan			
Kyung-Goo	Lee		MD	Myongji Hospital			
Moon-Soo	Lee		MD	Eulji University			
Myung Jae	Lee		MD	Jeonju Yeonlin Hospital			
Sang Eok	Lee		MD	Konyang University			
Sang Ho	Lee		MD	Kosin University			
Sang-Il	Lee		MD	Chunanam National University			
Si Hak	Lee		MD	Pusan National University			
Se Youl	Lee		MD	Chonbuk National University			
Seung Soo	Lee		MD	Kyungpook National University			
Sol	Lee		MD	Seoul Medical Center			
Yoontaek	Lee		MD	Ewha Womans University			
Seung Hyun	Lim		MD	Korea University			
Man-Sup	Lim		MD	Hallym University			
Jae Seok	Min		MD	Dongnam Institute of Radiological & Medical Sciences			
Sa-Hong	Kim		MD	University of Ulsan			
Woo-Kyun	Mok		MD	Yuhang Surgery Clinic			

Supplemental Online Content: Nonauthor Collaborators

*Indicates required information. Only first name, last name, and suffix will appear in PubMed.

***First Name and Middle Initial(s)**	***Last Name**	***Suffix (eg, Jr, III)**	Academic Degrees	Institution	Location (city, state/province, country)	Role or Contribution, eg, chair, principal investigator	Group (if more than 1 Group listed in the byline) and/or Subgroup (eg, Steering Committee)
Young-Jae	Mok		MD	Korea University			
Yoon-Hwan	Nam		MD	Hanil General Hospital			
Seung Jong	Oh		MD	Sungkyunkwan University			
Sung Don	Oh		MD	Inje University			
Sung Eun	Oh		MD	Sungkyunkwan University			
Sung Jin	Oh		MD	Inje University			
Cho Hyung	Park		MD	The Catholic University of Korea			
Dong Jin	Park		MD	University of Ulsan			
Ji Ho	Park		MD	Gyeongsang National University			
Ji Yeon	Park		MD	Kyungpook National University			
Ji Hyun	Park		MD	Seoul National University			
Jong-Min	Park		MD	National Medical Center			
Joong-Min	Park		MD	Chung-Ang University			
Ki Bum	Park		MD	Kyungpook National University			
Seong-Heum	Park		MD	Korea University			
Sungsoo	Park		MD	Korea University			
Yong Eun	Park		MD	Yeungnam University			
Young Suk	Park		MD	Seoul National University			
Chulkyu	Roh		MD	National Police Hospital			
Kun Ho	Roh		MD	Yonsei University			
Hoon	Ryu		MD	Yonsei University			
Seong Yeop	Ryu		MD	Chosun University			
Ho Seok	Seo		MD	The Catholic University of Korea			
Jeong Eun	Seo		MD	Hallym University			
Kyung Won	Seo		MD	Kosin University			
Sang Hyuk	Seo		MD	Inje University			
Won Jun	Seo		MD	Korea University			
Jang Won	Seon		MD	KS Hospital			

Supplemental Online Content: Nonauthor Collaborators

*Indicates required information. Only first name, last name, and suffix will appear in PubMed.

***First Name and Middle Initial(s)**	***Last Name**	***Suffix (eg, Jr, III)**	Academic Degrees	Institution	Location (city, state/province, country)	Role or Contribution, eg, chair, principal investigator	Group (if more than 1 Group listed in the byline) and/or Subgroup (eg, Steering Committee)
Dong Gue	Shin		MD	Seoul Red Cross Hospital			
Dong Woo	Shin		MD	Hallym University			
Ho-Jung	Shin		MD	Ajou University			
Tae Sung	Sohn		MD	Sungkyunkwan University			
Myoung Won	Son		MD	Soonchunhyang University			
Taeil	Son		MD	Gumdan Top Hospital			
Young-Gil	Son		MD	Keimyung University			
Geum Jong	Song		MD	Soonchunhyang University			
Jeong Ho	Song		MD	Ajou University			
Kyo Young	Song		MD	The Catholic University of Korea			
Sun-Kyo	Song		MD	Jaenam Hospital			
Byoung Jo	Suh		MD	Inje University			
Yun-Suhk	Suh		MD	Seoul National University			
Ji-Young	Sul		MD	Chunanam National University			
Choon Kyung	Sung		MD	Seoul Sacred Heart General Hospital			
Jun Young	Yang		MD	Gashon University			
Han Mo	Yoo		MD	The Catholic University of Korea			
Moon-Won	Yoo		MD	University of Ulsan			
Jeong-Hwan	Yook		MD	University of Ulsan			
Hong Man	Yoon		MD	National Cancer Center			
Ki Young	Yoon		MD	Kosin University			
Sang Il	Youn		MD	Chunanam National University			
Wansik	Yu		MD	Kyungpook National University			
