# Supplementary material for: Laparoscopic vs Open Distal Gastrectomy for Locally Advanced Gastric Cancer: 5-Year Outcomes of the KLASS-02 Randomized Clinical Trial
Source: JAMA Surg. 2022 Jul 20;157(10):879–86. doi: 10.1001/jamasurg.2022.2749 (PMC9301593; doi:10.1001/jamasurg.2022.2749)
Supplement: Supplement 4. — Data Sharing Statement [file jamasurg-e222749-s004.pdf]

Data Sharing Statement

Son. Laparoscopic vs Open Distal Gastrectomy for Locally Advanced Gastric Cancer. *JAMA Surg*. Published July 20, 2022. doi:10.1001/jamasurg.2022.2749

Data
Data available: No

Additional Information
Explanation for why data not available: Data is available only to participated researchers, because this was already agreed upon among participant researchers before the start of this trial.
